# Conserved Hypoxia-Responsive miRNA Programs Define Adaptive and Stress-Limiting Regulatory Axes in Hepatocellular Carcinoma

**DOI:** 10.3390/cells15151341

**Published:** 2026-07-26

**Authors:** Most Shumi Akhter Shathi, Mohammad Arif, Nobuhiro Nozaki, MD Nazmul Hasan, Yutaro Ide, Yoshiyuki Akiyama, Shaohsu Wang, Sirazul Islam, Tanjila Rahman, Tomohide Kuramoto, Yu Furusawa, Takeshi Sogawa, Kaori Takahashi, Aki Noguchi, Tatsuro Hifumi, Shinji Hirano, Noriaki Miyoshi, Osamu Yamato, Masashi Takahashi, Naoki Miura

**Affiliations:** 1 Joint Graduate School of Veterinary Medicine, Kagoshima University, Kagoshima 890-8580, Japan; shathi.vet@gmail.com (M.S.A.S.); mdarif38515@bau.edu.bd (M.A.); non.21non.21no.z@gmail.com (N.N.);; 2 Department of Microbiology and Hygiene, Bangladesh Agricultural University, Mymensingh 2202, Bangladesh; 3 Department of Livestock Services (DLS), Farmgate, Dhaka 1215, Bangladesh; 4 Department of Anatomy, Histology & Physiology, Sher-e-Bangla Agricultural University, Dhaka 1207, Bangladesh; 5 Veterinary Teaching Hospital, Joint Faculty of Veterinary Medicine, Kagoshima University, Kagoshima 890-8580, Japan; 6 Department of Veterinary Pathology, Joint Faculty of Veterinary Medicine, Kagoshima University, Kagoshima 890-8580, Japan

**Keywords:** hypoxia responsive miRNA, extracellular vesicles, circulating biomarkers, MAPK pathway, PI3K-Akt signaling, cross-species analysis

## Abstract

**Highlights:**

**What are the main findings?**

- Integrated miRNA profiling identified 11 hypoxia-responsive miRNAs in canine hepatocellular carcinoma, highlighting miR-210 and miR-34a as consistently upregulated candidates.
- Plasma extracellular vesicle-derived miR-210 and miR-34a showed excellent diagnostic performance for canine HCC and were associated with hypoxia-related signaling pathways, including MAPK, Ras, PI3K-Akt, and Rap1.

**What are the implications of the main findings?**

- Extracellular vesicle-associated miR-210 and miR-34a represent promising non-invasive biomarkers for the diagnosis of canine hepatocellular carcinoma.
- Cross-species conservation of hypoxia-responsive miRNA networks underscores the translational relevance of canine HCC to human disease.

**Abstract:**

Hypoxia-driven regulatory mechanisms play a critical role in tumor progression and therapeutic resistance in hepatocellular carcinoma (HCC), yet hypoxia-responsive microRNAs (HRMs) remain incompletely characterized. This study aimed to identify HRMs in canine HCC to evaluate their diagnostic potential and translational relevance to human disease. Next-generation sequencing of two canine HCC cell lines under normoxic and hypoxic conditions identified 332 and 321 differentially expressed miRNAs, respectively. Integrating these with tumor tissue data revealed 11 HRMs, featuring consistent upregulation of cfa-miR-210 and cfa-miR-34a, which was validated via RT-qPCR in hypoxic cells and clinical tissues. Both miRNAs were significantly elevated in plasma-derived extracellular vesicles (EVs), highlighting their value as promising circulating biomarkers (AUC 1.00 for miR-210; 0.98 for miR-34a). Target gene and pathway analyses identified shared regulatory nodes, including TGIF2 and SPRED1; and enrichment of MAPK, Ras, PI3K-Akt, and Rap1 signaling, broadly linked to hypoxia adaptation, cellular metabolism, and stress-response signaling. Cross-species comparison with human HCC datasets showed that, while miR-210 associates with poor prognosis in human HCC, miR-34a exhibits tumor-suppressive features. These findings define complementary HRM programs in canine HCC, reflecting conserved adaptive and stress-limiting regulatory mechanisms with potential diagnostic and translational relevance to human HCC.

## 1. Introduction

Hepatocellular carcinoma (HCC) is the most prevalent primary hepatic malignancy in both humans and dogs [1], and it accounts for more than 50% of such tumors in dogs [2]. When found in canine medicine, this malignant epithelial tumor of hepatocytic origin is most commonly diagnosed in older dogs [3].

HCC has a different incidence in humans and dogs, but the canine disease shares substantial pathological, molecular, and clinical features with its human counterpart, suggesting that canine HCC could serve as a spontaneous translational model in oncology [2,4,5]. While traditional rodent models provide important opportunities for controlled genetic and mechanistic studies, chemically induced tumors and xenograft models may not fully reproduce the heterogeneity and intact immune microenvironment of spontaneously arising human cancers. In contrast, companion dogs develop spontaneous hepatic malignancies in immunocompetent hosts and share environmental exposures with humans. Canine HCC may therefore serve as a valuable spontaneous model that complements traditional murine systems and supports the cross-species evaluation of candidate biomarkers and regulatory pathways [6]. Furthermore, both human and canine patients with HCC may only present with adverse clinical signs once the cancer has reached an advanced stage [7]. Such late diagnosis limits therapeutic options, and is associated with a poor prognosis. Thus, both species would greatly benefit from the identification of biomarkers that enable earlier diagnosis. Comparative molecular investigations represent a promising approach to identify liquid biomarkers that can support cross-species evaluation of candidate regulatory pathways and advance diagnostic strategies in both canine and human patients.

HCC shows one of the defining hallmarks of solid tumors: hypoxia. This is a state of reduced oxygen availability within the tumor microenvironment (TME) and arises from an imbalance between rapid tumor growth and inadequate vascularization. Cellular hypoxia occurs when partial oxygen pressure (pO_2_) falls below 5% [8], and can be classified as moderate at levels between 5% and 2%, or severe at levels below 2% [9]. As a hallmark of the HCC microenvironment, hypoxia actively drives tumor progression, confers therapeutic resistance, and maintains cancer stem-like properties [10,11,12,13]. Beyond its direct effects on tumor cells, hypoxia extensively remodels the TME by orchestrating complex crosstalk among tumor-associated macrophages, cancer-associated fibroblasts, endothelial cells, and other stromal components, thereby facilitating immune evasion and aberrant angiogenesis [12,13]. Furthermore, hypoxia promotes metastasis by inducing epithelial-to-mesenchymal transition (EMT), a critical process that enhances the invasive and migratory capacities of primary tumor cells [14].

Cellular adaptation to hypoxic stress is governed by complex transcriptional and post-transcriptional regulatory networks, among which microRNAs (miRNAs) play a pivotal role. miRNAs are small non-coding RNAs (20–24 nt) that regulate gene expression post-transcriptionally and influence key tumorigenic processes, including proliferation, apoptosis, invasion, and metastasis. Hypoxia markedly reshapes the miRNA landscape by generating ‘hypoxamiRs’, a subset of miRNAs whose expression changes in response to hypoxic conditions [15]. In this study, we collectively refer to miRNAs whose expression is altered under hypoxic conditions as hypoxia-responsive miRNAs (HRMs). Hypoxia-inducible factors (HIFs) activate specific HRMs by triggering epigenetic modifications (e.g., DNA demethylation), altering proteins involved in miRNA maturation, and modulating multiple signaling pathways [15,16]. Several miRNAs are highly conserved between dogs and humans, including the liver-enriched miR-122 and other conserved miRNAs such as miR-1 and miR-21 providing a molecular basis for comparative investigations of canine and human HCC [17,18,19].

Among HRMs, miR-210 has frequently been described as the “master HRMs”, and miR-34a plays a complex, context-dependent dual role in tumor suppression and progression. miR-210 was one of the first HRMs to be identified as a direct transcriptional target of HIF [20], and has reportedly been shown to promote angiogenesis [21], inflammation [22], EMT [23], tumor migration and invasion [24], to support stem-cell survival [25], and to trigger a shift from mitochondrial respiration to glycolysis [26], while inhibiting apoptosis [27,28] due to hypoxic response. Hypoxia may induce markedly robust levels of miR-210, with increases of 30- to 100-fold across multiple mammalian cell types [15]. miR-210 may also stabilize HIF1α expression by inhibiting degradation of the enzyme glycerol-3-dehydrogenase-like 1 [29]. Furthermore, with activation of HIF1α, hypoxic stress reduces DNA methyltransferases (DNMT) activity, leading to demethylation of a CpG island associated with the miR-210 coding region and a corresponding increase in miR-210 expression [30].

Unlike miR-210, miR-34a is considered a HIF-independent HRM and a direct transcriptional target of p53, which has been labeled as the ‘guardian of the genome’ [31]. miR-34a promotes apoptosis and cell-cycle arrest [32,33]. The p53 protein is encoded by the TP53 gene, and is one of the most frequently mutated tumor suppressors in human cancers. The p53 signaling pathway is often compromised during tumor development. Hypoxia is known to elevate p53 protein levels primarily through stabilization rather than enhanced transcription. It has been shown to accumulate progressively as oxygen levels decrease and hypoxia becomes more prolonged. Importantly, this accumulation is more pronounced under severe hypoxia, whereas mild hypoxia can stabilize HIF-1α without substantially activating p53 [34].

As a well-known p53 target, miR-34a contributes to the cellular response to hypoxic stress and plays an important role in limiting tumor progression. However, miR-34a expression is also negatively regulated under hypoxia. RNA interference targeting HIF-1α prevents hypoxia-induced downregulation of pri-miR-34a, demonstrating that HIF-1α directly suppresses miR-34a under low-oxygen conditions [14]. This facilitates the activation of the STAT3 pathway and other downstream pathways, which promote EMT and enhance tumor aggressiveness [14]. Thus, the interplay between p53-mediated activation and HIF-1α-dependent suppression determines miR-34a levels in hypoxic tumors, ultimately influencing whether the cell undergoes a protective tumor-suppressive response or shifts toward EMT and malignant progression. Beyond hypoxia, miR-34a is also responsive to a variety of cellular stressors including inflammatory stimuli [35], oxidative stress [36], fibrosis-associated signaling [37], and DNA damage [38], reflecting its broader role as an essential effector of stress-induced tumor suppression.

The insights outlined above largely stem from human studies, and the contribution of HRMs to canine tumors, including HCC, remains largely unexplored. Given the high evolutionary conservation of many miRNAs between dogs and humans, comparative transcriptomic analyses of hypoxia-driven miRNA regulation in canine HCC offer a valuable opportunity to uncover conserved molecular mechanisms with translational relevance.

In this study, we aimed to comprehensively characterize HRMs in canine HCC by integrating molecular profiling of hypoxia-exposed canine HCC cell lines, clinical tumor tissues, and circulating compartments, and by comparing these with human datasets. Specifically, we identified conserved HRMs, including miR-210 and miR-34a, which may represent complementary regulatory patterns associated with cellular adaptation and stress-limiting responses to hypoxia. We further evaluated their clinical relevance and translational potential. Collectively, this study provides a comparative framework for hypoxia-driven miRNA regulation in canine hepatocellular carcinoma and highlights its potential translational significance.

## 2. Materials and Methods

### 2.1. Clinical Samples

Clinical specimens were obtained from 19 individual canine HCC patients, including liver tissue from 12 animals and plasma samples from 7 animals. All HCC diagnoses were confirmed histopathologically by a specialized pathologist and undergoing surgical treatment at the Veterinary Teaching Hospital of Kagoshima University (KUVTH) or affiliated veterinary clinics. Informed consent was obtained from each dog owner. The study donors comprised dogs of both sexes and multiple breeds and mixed ancestry, with an age range of 4–14 years.

Normal liver tissue samples were donated by Shin Nippon Biomedical Laboratories, Ltd., Drug Safety Research Laboratories (SNBL DSR, Kagoshima, Japan). The samples were originally collected from five healthy, purpose-bred Beagles [17]. These dogs had been purpose bred, and housed under controlled laboratory conditions for research unrelated to this study. The tissue samples were collected as part of that unrelated research, after the dogs had been sacrificed in accordance with the standard operating procedures and with the approval of the Institutional Animal Care and Use Committee of SNBL, as described previously [17]. Following excision, liver tissues were immersed in RNAlater for RNA stabilization, and then stored. The samples were shipped to the laboratory at the time of donation under temperature-controlled conditions. Detailed sample information is summarized in Table S1.

Blood samples used as controls were obtained from healthy canine donors free of tumors (*n* = 7) at an affiliated clinic (Chuou Aiken Animal Hospital, Kagoshima, Japan) as part of routine examinations. After collection, the blood samples from dogs with HCC (*n* = 7) and healthy controls (*n* = 7) were centrifuged at 3000× *g* for 10 min to separate plasma, followed by a second centrifugation at 16,000× *g* for 20 min to further remove cellular debris. The supernatant was carefully isolated to prevent pellet contamination. Both tissue and plasma samples were maintained at −80 °C until downstream analysis. All animal-related procedures were approved by the Ethics Committee for Veterinary Clinical Research of the Joint Faculty of Veterinary Medicine, Kagoshima University (Approval Nos. KVH220001 and KVH240013) and were conducted in accordance with the regulations and guidelines of the KUVTH. No animals were subjected to additional procedures solely for this study, and all samples were obtained during routine clinical evaluations or diagnostic procedures. A subset of the clinical samples included in this study partially overlapped with archived canine HCC samples used in previous studies from our group [39]; however, the present study addressed a distinct biological question and involved independent analyses focused on hypoxia-responsive miRNAs and EV-associated biomarkers. Previously reported canine HCC tissue miRNA profiles used for integrative comparison are cited in the relevant sections.

### 2.2. Cell Culture

Two well-characterized canine hepatocellular carcinoma (HCC) cell lines, 95-1044 (high proliferative capacity) and 95-0112 (low proliferative capacity), were used in this study as reported previously [39,40]. Cells preserved in CultureSure freezing medium (Wako Pure Chemical Industries, Ltd., Osaka, Japan) under liquid nitrogen were thawed and maintained under either normoxic (21% O_2_, 5% CO_2_, 37 °C) or hypoxic (2% O_2_, 5% CO_2_, 37 °C) conditions using a Heracell^TM^ CO_2_ incubator (Thermo Fisher Scientific, Waltham, MA, USA). Cells were cultured in Dulbecco’s modified Eagle’s medium (DMEM, Sigma-Aldrich, St. Louis, MO, USA) supplemented with 5% fetal bovine serum (Thermo Fisher Scientific, Waltham, MA, USA), 5% L-glutamine (Sigma-Aldrich, St. Louis, MO, USA), and 3.5 μg/mL spectinomycin (Sigma-Aldrich, St. Louis, MO, USA). Cell lysates were collected 48 h after initial culture, at approximately 80% confluency. Cell counting was performed using an automated cell counter (LUNA II, Logos Biosystems by Aligned Genetics, Inc., Seoul, Republic of Korea). Following collection, lysates were either stored at −80 °C for further use or processed immediately for RNA isolation.

### 2.3. Extracellular Vesicle Isolation

Plasma-derived extracellular vesicles (EVs) used in the present study were previously isolated [39] from the plasma of clinical canine HCC patients and healthy control dogs using the Total Exosome RNA and Protein Isolation Kit (Invitrogen, Thermo Fisher Scientific, Waltham, MA, USA) according to the manufacturer’s instructions. Briefly, 300 µL of plasma was diluted with half a volume of 1× PBS, followed by the addition of 90 µL of Exosome Precipitation Reagent (Invitrogen, Thermo Fisher Scientific). After thorough vortexing, the mixture was incubated at room temperature for 10 min and centrifuged at 10,000× *g* for 5 min. After careful aspiration of the supernatant, the EV pellet was resuspended in 150 µL of 1× PBS and stored at −80 °C until further use. These previously isolated plasma-derived EVs were subsequently used to analyze the expression of the targeted HRMs in the present study. In our previous study, the same EV preparations were evaluated by Western blotting for the presence of the EV-associated markers CD9 and TSG101 [39].

### 2.4. RNA Extraction and Small RNA Sequencing

Total RNA was extracted from lysates of cultured cells and liver tissues using the mirVana^TM^ miRNA Isolation Kit (Thermo Fisher Scientific, Waltham, MA, USA). RNA was isolated from plasma and plasma-derived EVs using the mirVana^TM^ PARIS Kit and the Total Exosome RNA and Protein Isolation Kit (Thermo Fisher Scientific, Waltham, MA, USA), following the manufacturers’ protocols and the procedure described previously [39]. RNA concentration was determined using a NanoDrop 2000c spectrophotometer (Thermo Fisher Scientific, Waltham, MA, USA). RNA integrity and quality were evaluated with an Agilent 2100 Bioanalyzer (Agilent Technologies, Santa Clara, CA, USA), with a mean RNA integrity number (RIN) of 8.5 (range: 7.5–9.6). Electropherograms consistently displayed a distinct peak corresponding to small RNAs (~25 nt), confirming the samples’ suitability for subsequent small RNA-seq analysis.

Cell samples from two canine HCC cell lines (95-1044 and 95-0112) were cultured under normoxic and hypoxic conditions (three samples in each group) and then underwent next-generation sequencing (NGS). Total RNA was extracted from each relevant sample and sent for analysis at a specialist laboratory (Hokkaido System Science, Sapporo, Japan) to prepare the RNA libraries and sequence data using an Illumina HiSeq 2500 (Illumina Inc., San Diego, CA, USA) platform as described previously [39].

### 2.5. Bioinformatics and Differential Expression Analysis

Raw sequencing data were processed using CLC Genomics Workbench v24.0 (Qiagen Digital Insights, CLC Bio, Qiagen, Germany), following the developer’s instructions. Adapter sequences were trimmed, and low-quality and ambiguous reads were removed. The remaining reads were annotated against miRBase v22.1 using *Canis lupus familiaris* and *Homo sapiens* as reference species. Because canine miRNA annotation remains incomplete, human miRBase sequences were additionally used to identify potentially conserved miRNAs that may not yet be represented in current canine miRNA databases. Differentially expressed miRNAs (DE miRNAs) were identified using the following thresholds: |fold change| > 2, false discovery rate (FDR) *p* < 0.05, and maximum group mean > 10. Principal component analysis (PCA), volcano plots, and heatmaps were generated using CLC Genomics Workbench to visualize the annotated miRNAs.

### 2.6. Quantification of miRNA Expression by RT-qPCR

The relative expression of selected miRNAs was evaluated in tissues, cell lines, plasma and plasma-derived EVs by RT-qPCR, as previously described [39]. Briefly, 1.25 μL of total RNA (2 ng/μL for tissues and cell samples) was reverse transcribed into cDNA using the TaqMan MicroRNA Reverse Transcription Kit (Thermo Fisher Scientific, Waltham, MA, USA) in a T100 thermal cycler (Bio-Rad Laboratories, Hercules, CA, USA). qPCR was performed on a QuantStudio 3 real-time PCR system (Thermo Fisher Scientific, Waltham, MA, USA) using TaqMan Fast Advanced Master Mix (Thermo Fisher Scientific, Waltham, MA, USA). Thermal cycling conditions consisted of 50 °C for 2 min and 95 °C for 20 s, followed by 40 cycles of 95 °C for 1 s and 60 °C for 20 s, according to the manufacturer’s instructions. All assays were performed in at least two independent replicates. Relative miRNA expression was calculated using the 2^−ΔΔCT^ method and normalized to appropriate endogenous controls: RNU6B (TaqMan MicroRNA Assay ID: 001093) for tissue and cell samples, miR-16 (ID: 000391) for plasma samples, and miR-186 (ID: 002285) for plasma-derived EVs [39].

### 2.7. Putative Target Gene Prediction and Network Analysis

Potential target genes of cfa-miR-210 and cfa-miR-34a were predicted using three independent miRNA target prediction algorithms: TargetScan v8.0 (https://www.targetscan.org/; accessed 17 July 2025), miRDB (http://mirdb.org/cgi-bin/search.cgi; accessed 17 July 2025) and miRWalk (http://mirwalk.umm.uni-heidelberg.de/; accessed 17 July 2025). Because individual prediction tools use different algorithms and may generate false-positive results, overlapping candidate genes identified by multiple databases were considered high-confidence predicted miRNA targets to improve prediction reliability. To further enhance reliability while avoiding the exclusion of potentially important candidates, moderate filtering thresholds were applied: TargetScan (context++ score ≤− 0.15), miRDB (target score ≥ 60), and miRWalk (binding probability ≥ 0.9, energy ≤− 20). Finally, miRNA-predicted target gene interaction networks were constructed using Cytoscape v3.10.3.

### 2.8. Functional Annotation and Pathway Enrichment Analysis

The Database for Annotation, Visualization and Integrated Discovery (DAVID) Bioinformatics Resources (https://davidbioinformatics.nih.gov/; accessed 17 July 2025) was used for functional annotation including Gene Ontology (GO) terms and Kyoto Encyclopedia of Genes and Genomes (KEGG) pathways. Candidate target genes predicted by all three algorithms were considered for functional annotation.

### 2.9. Human Dataset and Cross-Species Translational Analysis

Targeted miRNAs were assessed for their expression and prognostic value in human HCC using publicly available TCGA-based online platforms, including ENCORI (https://rnasysu.com/encori/; accessed 22 July 2025), KM plotter (https://kmplot.com/analysis/; accessed 22 July 2025), and UALCAN (https://ualcan.path.uab.edu/; accessed 22 July 2025). To independently validate the differential expression patterns of human homologs of candidate HRMs in human HCC, an additional dataset (GSE21362, *n* = 73 paired tumor and non-tumor liver tissues) was analyzed from the NCBI Gene Expression Omnibus (GEO) repository (https://www.ncbi.nlm.nih.gov/geo/query/acc.cgi?acc=GSE21362; accessed 14 July 2026). Normalized expression values for hsa-miR-34a and hsa-miR-210 were extracted, and paired two-tailed Student’s *t*-tests were performed to determine differential expression between paired human HCC tumor and adjacent non-tumoral liver tissues.

### 2.10. Statistical Analysis

Statistical analyses were performed according to data distribution and variance. Normality was assessed using the Shapiro–Wilk test. For normally distributed data, comparisons between two groups, including miRNA expression levels and miRNA mapping rates under hypoxic and normoxic conditions, were performed using unpaired two-tailed Student’s *t*-tests. When unequal variances were identified by Levene’s test, Welch’s correction was applied. Non-normally distributed data were analyzed using the non-parametric Mann–Whitney U test. Correlations among NGS samples were assessed using Spearman’s rank correlation coefficient. Receiver Operating Characteristic (ROC) curve analysis was performed to assess the diagnostic performance of individual miRNAs, and the area under the curve (AUC) was calculated using the Wilson–Brown method. *p*-values < 0.05 were considered statistically significant, with significance levels defined as follows: *p* < 0.05 (*), *p* < 0.01 (**), *p* < 0.001 (***). All statistical analyses and data visualizations were performed using GraphPad Prism v8.0 (GraphPad Software, San Diego, CA, USA).

### 2.11. Use of Artificial Intelligence Tools

ChatGPT (GPT-4o, OpenAI) was used during manuscript preparation to improve linguistic clarity, refine sentence structure, and assist in the preparation of the graphical abstract. All outputs generated by the tool were reviewed, edited, and verified by the authors. The authors take full responsibility for the accuracy and integrity of the manuscript. Artificial intelligence was not used for study design, data collection, data analysis, results generation, or scientific interpretation.

## 3. Results

### 3.1. Hypoxia Enhances Canonical miRNA Expression in Canine HCC

**Figure 1:**
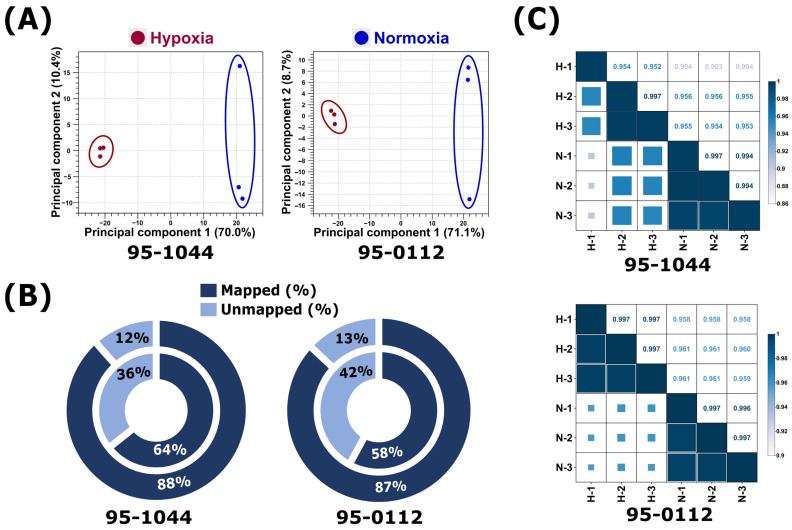
Hypoxia induces widespread alterations in canonical miRNA expression within canine HCC cell lines. (**A**) Principal component analysis (PCA) of miRNA expression profiles generated by next-generation sequencing of two canine hepatocellular carcinoma (HCC) cell lines, 95-1044 (**left**) and 95-0112 (**right**), cultured under normoxic (*n* = 3 per cell line) and hypoxic (*n* = 3 per cell line) conditions. Each axis shows the proportion of total data variability captured by that principal component. Blue denotes normoxic samples and red denotes hypoxic samples. (**B**) Proportional distribution of sequencing reads mapped to annotated miRNAs under normoxic (inner ring) and hypoxic (outer ring) conditions. The left donut plot represents data from 95-1044 cells, while the right donut plot represents data from 95-0112 cells. (**C**) Sample-to-sample correlation heatmaps between normoxic and hypoxic conditions for each cell line (**upper** panel, 95-1044; **lower** panel, 95-0112).

Next-generation sequencing was performed on total RNA from two HCC cell lines (95-1044 and 95-0112) cultured under normoxic and hypoxic conditions. Data were analyzed using CLC Genomics Workbench with both canine and human miRBase references to facilitate the identification of potentially conserved miRNAs. Clean reads showed a characteristic miRNA length distribution of 20–24 nt across all samples (Figure S1). Principal component analysis (PCA) demonstrated clear separation between hypoxic and normoxic samples in both cell lines, indicating hypoxia-dependent global miRNA expression changes (Figure 1A). Consistently, hypoxia markedly increased the proportion of reads mapped to known miRNAs. In the 95-1044 cell line, miRNA mapping increased from 64% under normoxia to 88% under hypoxia, while in the 95-0112 cell line, mapping increased from 58% to 87% (Figure 1B and Table S2). These increases were statistically significant (unpaired *t*-test, *p* < 0.001), suggesting enhanced expression or stability of canonical miRNAs under hypoxic stress. Furthermore, sample-to-sample correlation analysis demonstrated high reproducibility within experimental groups and clear segregation between hypoxic and normoxic conditions in both cell lines (Figure 1C). Collectively, these results confirm robust data quality and demonstrate that hypoxia induces coordinated and reproducible changes in canonical miRNA expression in canine HCC cells.

### 3.2. Differentially Expressed miRNAs Under Hypoxia Reveal Limited Annotation Coverage in the Canine miRNA Database

**Figure 2:**
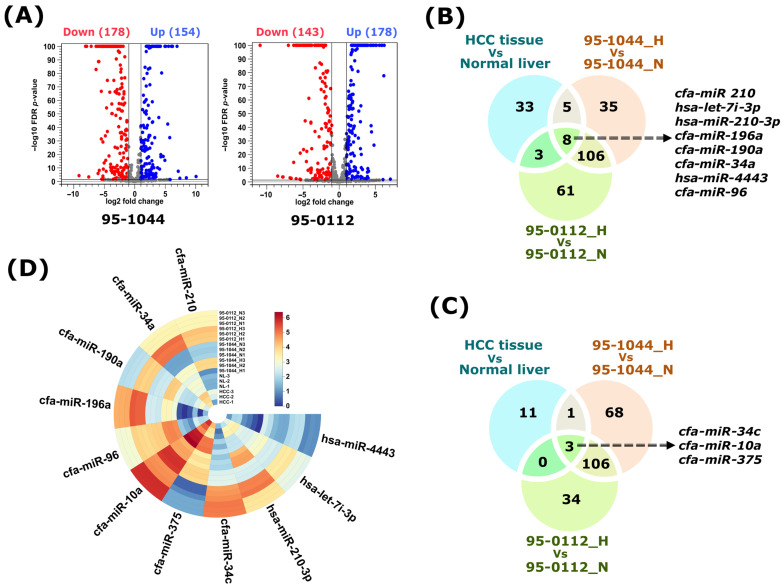
Identification of hypoxia-responsive miRNAs in canine hepatocellular carcinoma. (**A**) Volcano plots showing differentially expressed miRNAs between hypoxic and normoxic conditions in canine HCC cell lines 95-1044 (**left**) and 95-0112 (**right**). Differential expression was defined using an FDR-adjusted *p* < 0.05, |fold change| > 2, and a minimum expression threshold (maximum group mean > 10). Red and blue dots represent significantly downregulated and upregulated miRNAs, respectively, while gray dots denote those without significant changes. (**B**,**C**) Venn diagrams (represented by different colored circles for each comparison) illustrating the overlap of hypoxia-responsive miRNAs (HRMs) that were consistently upregulated (**B**) or downregulated (**C**) across hypoxic conditions in both cell lines and canine HCC clinical tissues. Numbers represent unique miRNA family names to minimize redundancy arising from multiple mature miRNAs derived from the same family. (**D**) Circular heatmap depicting the expression patterns of selected HRMs across NGS datasets from normoxic and hypoxic cell lines as well as canine HCC clinical tissues. Color intensity ranging from blue (lower expression) to red (higher expression) represents relative expression levels.

Following miRNA annotation, differential expression analysis was performed to compare hypoxic and normoxic HCC cells using the criteria: |fold change| > 2, FDR-adjusted *p* < 0.05, and a minimum expression threshold (maximum group mean > 10). In 95-1044 and 95-0112 cells, 332 (154 upregulated, 178 downregulated) and 321 (178 upregulated, 143 downregulated) HRMs were identified, respectively, based on comparison with the relevant normoxic cells (Figure 2A and Tables S3 and S4). Heatmap clustering demonstrated distinct expression profiles between hypoxic and normoxic conditions (Figure S2).

To assess species-specific annotation coverage of hypoxia-regulated miRNAs, we examined their annotation using both canine and human miRBase references. For this analysis, miRNAs were collapsed into unique miRNA family names to minimize redundancy arising from multiple mature miRNAs derived from the same family. Consequently, the overall count of significantly altered miRNAs decreased (267 in 95-1044 and 250 in 95-0112) compared with the previously reported counts based on individual miRNA species. Using this family-level approach, a substantial proportion of hypoxia-responsive miRNAs were annotated exclusively from the human miRNA database. In the 95-1044 cell line, 33.05% (40/121) of upregulated and 48.63% (71/146) of downregulated miRNA families were annotated exclusively from the human miRNA database, while corresponding proportions of 34.55% (47/136) and 55.26% (63/114) were observed in the 95-0112 cell line (Figure S3). Collectively, these results indicate that a considerable fraction of hypoxia-responsive miRNAs in canine HCC may not yet be fully represented in current canine miRNA databases.

### 3.3. Hypoxic Stimuli Consistently Dysregulate miRNA Expression in Canine HCC

To identify HRMs relevant to canine HCC, we conducted a Venn diagram analysis comparing differentially expressed miRNAs with concordant expression trends in two hypoxia-exposed HCC cell lines (95-1044 and 95-0112) and previously reported miRNAs from canine HCC clinical tissues (Table S5) [39]. With this approach, we aimed to prioritize miRNAs that were consistently dysregulated under hypoxic conditions in vitro and in HCC tissues in vivo, thereby identifying HCC-associated hypoxia-responsive candidates. The intersection of the three datasets revealed 11 HRMs, comprising eight upregulated and three downregulated miRNAs (Figure 2B,C). Among the upregulated HRMs, five were annotated using the canine reference (cfa-miR-210, cfa-miR-196a, cfa-miR-190a, cfa-miR-34a, and cfa-miR-96), whereas three were identified as human-reference annotated candidate miRNAs (hsa-let-7i-3p, hsa-miR-210-3p, and hsa-miR-4443). The downregulated HRMs included cfa-miR-34c, cfa-miR-10a, and cfa-miR-375. Heatmap analysis illustrated the expression patterns of these HRMs across all NGS datasets (Figure 2D). Based on their consistent differential expression across NGS analyses (Figures S4 and S5), a subset of canine-specific miRNAs, including cfa-miR-210, cfa-miR-34a, cfa-miR-190a, cfa-miR-96, and cfa-miR-10a, was selected for downstream validation.

### 3.4. qPCR Validation Identifies cfa-miR-210 and cfa-miR-34a as Robust HRMs in Canine HCC

**Figure 3:**
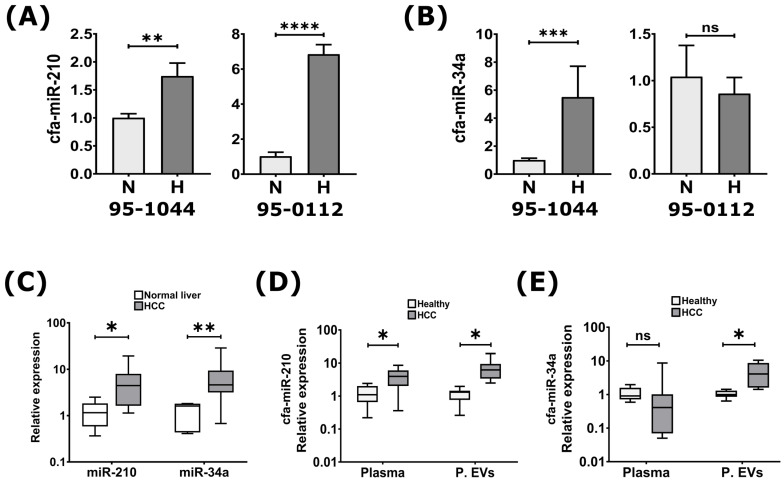
qPCR validation of hypoxia-responsive miRNAs in canine HCC cells, clinical tissues, and circulating compartments. (**A**) Relative expression of cfa-miR-210 measured by RT-qPCR in canine HCC cell lines 95-1044 (**left**) and 95-0112 (**right**) following hypoxic exposure, normalized to RNU6B (*n* = 6 per group). (**B**) Relative expression of cfa-miR-34a in 95-1044 (left) and 95-0112 (right) cells under hypoxic conditions, normalized to RNU6B (*n* = 6 per group). White bars represent normoxia (N) and dark gray bars represent hypoxia (H). (**C**) Relative expression levels of cfa-miR-210 and cfa-miR-34a in canine HCC clinical tissues (dark gray boxes; *n* = 12) compared with normal liver controls (white boxes; *n* = 5), plotted on a log10 scale. (**D**) Circulating expression levels of cfa-miR-210 in plasma and plasma-derived EVs (P. EVs) from canine HCC patients (dark gray boxes; *n* = 7) and healthy controls (white boxes; *n* = 7), plotted on a log10 scale. (**E**) Circulating expression levels of cfa-miR-34a in plasma (*n* = 7) and plasma-derived EVs (*n* = 6), plotted on a log10 scale. Data are summarized as mean ± SD. Statistical comparisons between normally distributed datasets were performed using an unpaired two-tailed Student’s *t*-test, whereas the Mann–Whitney *U* test was applied to non-normally distributed datasets. Statistical significance was defined as *p* < 0.05 (∗, *p* < 0.05; ∗∗, *p* < 0.01; ∗∗∗, *p* < 0.001; ∗∗∗∗, *p* < 0.0001; ns, not significant).

To verify the reliability of the NGS results, we examined the expression levels of five selected HRMs by qPCR at 48 h under hypoxic conditions in 95-1044 and 95-0112 cells, corresponding to the time point used for NGS analysis. Aligning with our sequencing results, cfa-miR-210 expression was significantly increased under hypoxia in both cell lines (*p* < 0.05; Figure 3A). cfa-miR-34a showed a significant upregulation in the highly proliferative 95-1044 cells, whereas no significant difference (*p* > 0.05) was observed in the slowly proliferative 95-0112 cells (Figure 3B). The remaining selected HRMs did not exhibit significant expression changes under hypoxic conditions in either cell line (Figure S6A–C). To assess clinical relevance, we further quantified the expression of the selected HRMs in HCC tissues from 12 canine patients using TaqMan-based qPCR. In agreement with the in vitro findings, cfa-miR-210 and cfa-miR-34a were significantly upregulated in canine HCC tissues (*p* < 0.05; Figure 3C). In contrast, most other HRMs showed no significant differences in expression (*p* > 0.05), except for cfa-miR-10a, which was significantly downregulated in clinical samples (*p* < 0.05; Figure S6D). Collectively, these results validate the NGS findings and highlight cfa-miR-210 and cfa-miR-34a as consistently hypoxia-responsive miRNAs in canine HCC, supporting their potential biological relevance under hypoxic stress.

### 3.5. Plasma-Derived EV Associated cfa-miR-210 and cfa-miR-34a Demonstrate Superior Diagnostic Performance

**Figure 4:**
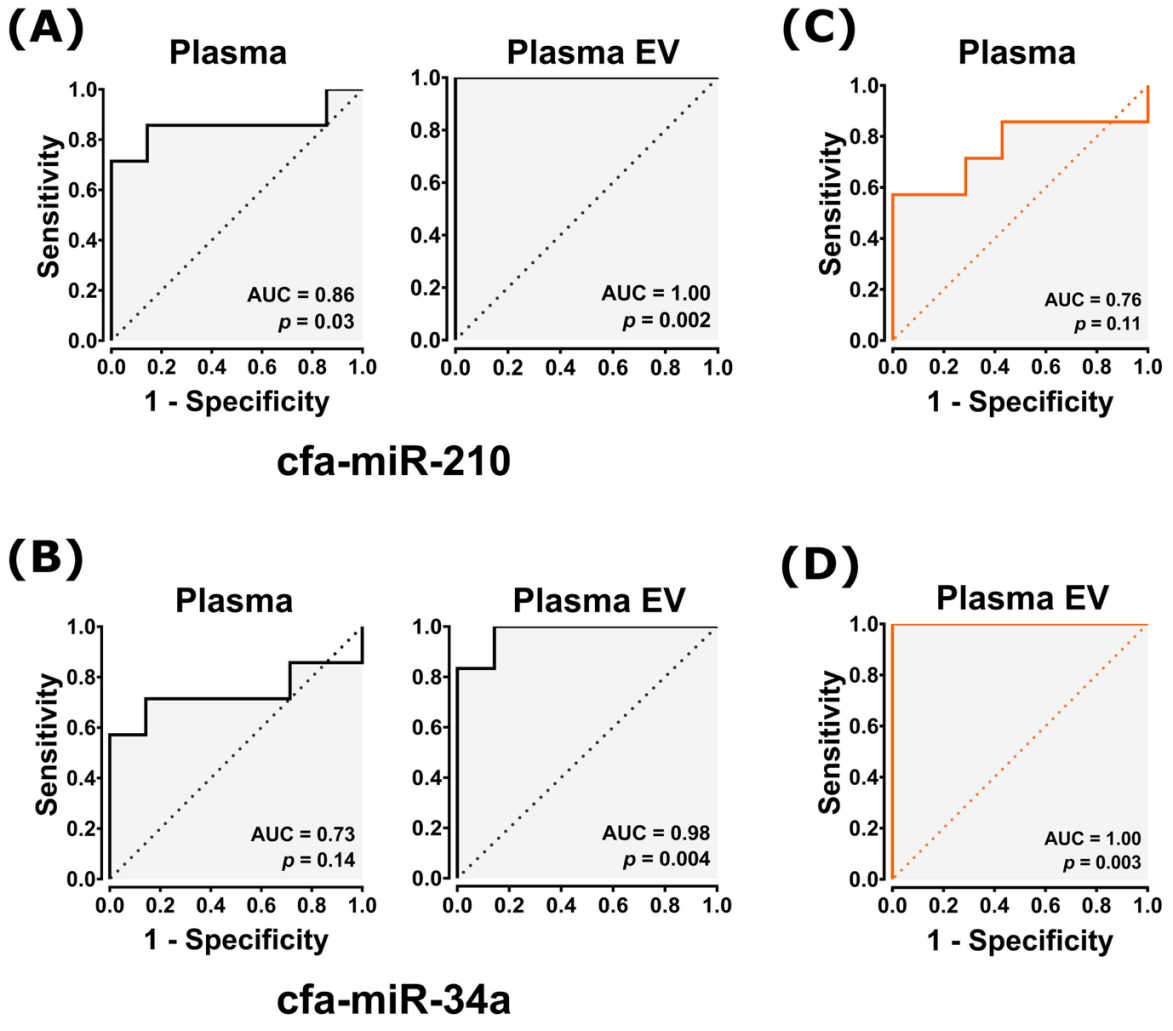
Diagnostic performance of hypoxia-responsive miRNAs in plasma and plasma-derived EVs. (**A**) Receiver operating characteristic (ROC) analysis of cfa-miR-210 expression in plasma (**left**; AUC = 0.86, *p* = 0.03) and plasma-derived EVs (**right**; AUC = 1.00, *p* = 0.002) from canine HCC patients. (**B**) ROC analysis of cfa-miR-34a expression in plasma (**left**; AUC = 0.73, *p* = 0.14) and plasma-derived EVs (**right**; AUC = 0.98, *p* = 0.004). (**C**,**D**) Combined ROC analysis based on the average expression of cfa-miR-210 and cfa-miR-34a in plasma ((**C**); AUC = 0.76, *p* = 0.11) and plasma-derived EVs ((**D**); AUC = 1.00, *p* = 0.003). The orange line represents the combined diagnostic model based on the average expression of cfa-miR-210 and cfa-miR-34a, whereas the diagonal dashed line indicates the performance expected by random chance (AUC = 0.5) and serves as the reference for evaluating diagnostic discrimination. ROC curve analysis was performed to evaluate diagnostic performance, with the area under the curve (AUC) used as the summary measure. The Wilson–Brown method was applied to calculate 95% confidence intervals for sensitivity and specificity. Statistical significance was established at *p* < 0.05. All ROC analyses were carried out using GraphPad Prism v8.0.

To evaluate the circulating biomarker potential of hypoxia-responsive miRNAs, the expression levels of cfa-miR-210 and cfa-miR-34a were quantified by RT-qPCR in plasma and plasma-derived EVs from canine HCC patients and healthy controls. cfa-miR-210 was significantly upregulated in both plasma (*p* < 0.05) and plasma-derived EVs (*p* < 0.01), indicating a consistent elevation across circulating compartments (Figure 3D). In contrast, cfa-miR-34a showed significant upregulation exclusively in plasma-derived EVs (*p* < 0.05), while no significant difference was observed in total plasma, suggesting compartment-specific enrichment (Figure 3E). ROC curve analysis demonstrated strong diagnostic performance of cfa-miR-210, with an area under the curve (AUC) of 0.86 (*p* < 0.05) in plasma and 1.00 (*p* < 0.01) in plasma-derived EVs (Figure 4A). Similarly, cfa-miR-34a exhibited excellent discriminatory power in plasma-derived EVs (AUC = 0.98, *p* < 0.01), whereas its diagnostic accuracy in plasma was limited (AUC = 0.73, *p* > 0.05) (Figure 4B). These results indicate that plasma-derived EV-associated miRNAs may provide superior diagnostic resolution to total plasma miRNAs.

To explore whether combined miRNA evaluation improves diagnostic performance, ROC analyses were performed using the average expression values of cfa-miR-210 and cfa-miR-34a. The combined miRNA signature showed moderate diagnostic accuracy in plasma (AUC = 0.76; Figure 4C) and achieved optimal discrimination in plasma-derived EVs (AUC = 1.00; Figure 4D). Collectively, these findings highlight the potential utility of circulating EV-associated cfa-miR-210 and cfa-miR-34a, individually and in combination, as non-invasive biomarkers for canine HCC.

### 3.6. Functional Enrichment Analysis of Predicted cfa-miR-210 and cfa-miR-34a Target Candidates Reveals Potential Oncogenic Pathways in Canine HCC

**Figure 5:**
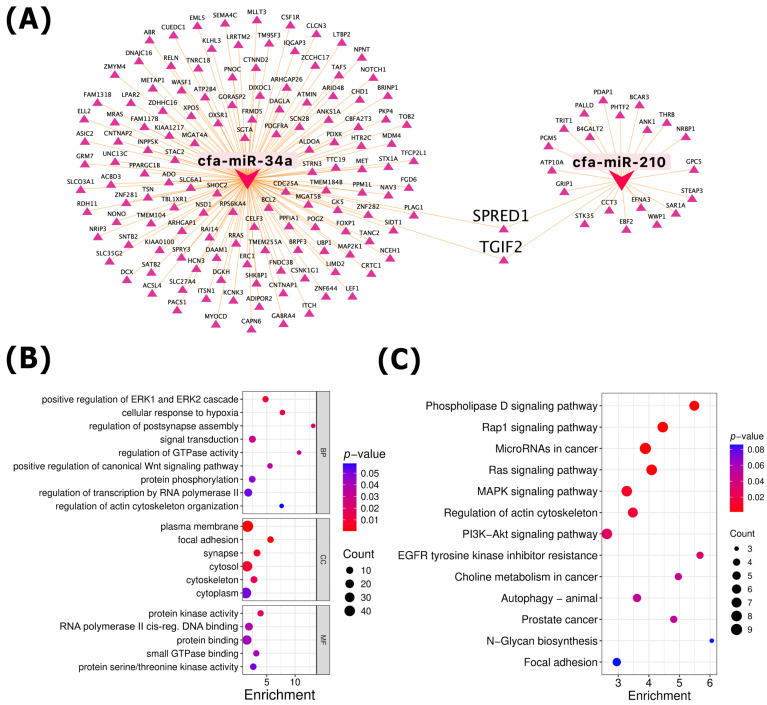
Integrative miRNA-mRNA network and functional enrichment analysis of predicted cfa-miR-210 and cfa-miR-34a target candidates in canine HCC. (**A**) In silico miRNA-mRNA interaction network generated using Cytoscape v3.10.3 based on a total of 154 high-confidence candidate target genes of cfa-miR-210 and cfa-miR-34a commonly predicted by TargetScan, miRDB, and miRWalk. (**B**) Gene Ontology (GO) enrichment analysis of the combined predicted candidate gene set (*n* = 154), showing significantly enriched biological processes, molecular functions, and cellular components. (**C**) KEGG pathway enrichment analysis of the same candidate gene set. For both GO term and KEGG pathways, dot size corresponds to the number of associated genes, whereas dot color reflects the level of statistical significance. Terms with *p* < 0.05 were considered statistically significant.

To explore putative downstream molecular mechanisms potentially associated with cfa-miR-210 and cfa-miR-34a in canine HCC, we performed integrative target prediction using three established databases: TargetScan, miRDB, and miRWalk. For cfa-miR-210, 968, 47, and 8826 predicted candidate targets were identified with the respective databases, whereas the corresponding figures for cfa-miR-34a were 643, 638, and 9072 putative targets (Table S6). Intersection analysis across all three databases identified 20 high-confidence candidate targets for cfa-miR-210 and 134 for cfa-miR-34a (Figure S7). These putative target genes were visualized using a miRNA-mRNA interaction network constructed in Cytoscape v3.10.3 (Figure 5A), which suggested TGIF2 and SPRED1 as predicted shared target candidates of both miRNAs. These putative candidates warrant further experimental validation.

To explore the functional relevance of the combined candidate gene set (*n* = 154, Table S7), gene ontology (GO) and KEGG pathway enrichment analyses were conducted using DAVID. GO analysis revealed significant enrichment in biological processes related to signal transduction, hypoxia response, transcriptional regulation, and Wnt and ERK1/ERK2 signaling. The enriched molecular functions were mainly related to kinase activity, GTPase binding, and RNA polymerase II interaction, while cellular components were primarily associated with the plasma membrane, focal adhesion, cytoskeleton, and cytosol (Figure 5B and Table S8). KEGG pathway analysis demonstrated significant enrichment of key oncogenic signaling pathways, including MAPK, Ras, PI3K-Akt, Rap1 signaling, and microRNAs in cancer (Figure 5C and Table S9). Together, these findings indicate that cfa-miR-210 and cfa-miR-34a converge on conserved oncogenic networks involved in cell proliferation, survival, and migration, supporting the view that they play a linked regulatory role in hypoxia-driven canine HCC progression.

### 3.7. Hypoxia-Responsive miR-210 and miR-34a Are Evolutionarily Conserved Between Canine and Human HCC

To assess the evolutionary conservation of hypoxia-responsive miRNAs identified in canine HCC, we compared mature miRNA sequences between dog and human genomic data using the miRBase database. This analysis demonstrated that cfa-miR-210 is highly conserved with hsa-miR-210-3p, and that cfa-miR-34a aligns closely with hsa-miR-34a-5p, indicating potential conservation of biological function across the species (Figure S8).

### 3.8. miR-34a, but Not miR-210, Shows Inverse Correlation with Shared Targets TGIF2 and SPRED1 in Human HCC

To explore potential regulatory interactions between these conserved miRNAs and their predicted targets, we utilized The Encyclopedia of RNA Interactomes (ENCORI) database. Correlation analysis revealed a weak positive association of hsa-miR-210-3p with TGIF2 and SPRED1, whereas hsa-miR-34a-5p exhibited weak negative associations with both predicted target genes (Figure S9). Despite the modest correlation strength, the negative relationship observed for miR-34a-5p aligns with the canonical mechanism of miRNA-mediated mRNA repression.

### 3.9. miR-34a Is Consistently Upregulated Across Independent Human HCC Cohorts

To evaluate the translational relevance of candidate HRMs in human HCC, we assessed their expression across multiple independent human HCC datasets. Initial analysis using the UALCAN and ENCORI databases (derived from TCGA-LIHC) revealed no dysregulation of hsa-miR-210-3p (*p* > 0.05), whereas hsa-miR-34a-5p exhibited significant upregulation in human HCC tissues compared to non-tumoral liver controls, mirroring the elevated levels observed in canine HCC tissues and hypoxia-exposed cells (Figure S10A–D).

To independently validate these cross-species expression patterns, we interrogated an additional GEO dataset (GSE21362). Consistent with our TCGA and canine findings, hsa-miR-34a was significantly upregulated (*p* < 0.05) in paired tumor tissues. Conversely, hsa-miR-210 showed no significant (*p* > 0.05) differential expression between paired tumor and non-tumoral liver tissues (Figure S10E,F), confirming the divergence in miR-210 expression patterns between canine and human HCC cohorts. The complete normalized expression values of the candidate HRMs are provided in Table S10.

### 3.10. miR-210 and miR-34a Exhibit Opposing Prognostic Associations in Human HCC

To assess the clinical significance of the hypoxia-responsive miRNAs identified in canine HCC, we performed prognostic analysis of their human counterparts using ENCORI and KMplotter datasets. High expression of hsa-miR-210-3p was consistently associated with significantly poorer overall survival in HCC patients (ENCORI: log-rank *p* = 0.00011, HR = 2.00; KMplotter: log-rank *p* = 1.9 × 10^−6^, HR = 2.34) (Figure S11A,C). This aligns with the established role of miR-210 as an HRM linked to aggressive tumor behavior. In contrast, hsa-miR-34a-5p displayed a prognostic pattern in which higher expression correlated with improved survival in KM plotter (log-rank *p* = 0.0011, HR = 0.56) analysis, although the similar trend in analysis with the ENCORI did not reach statistical significance (Figure S11B,D). Together, these findings indicate that both miRNAs retain clinically relevant prognostic value in human HCC, supporting the translational applicability of the hypoxia-related miRNA signatures identified in the canine model.

## 4. Discussion

Inadequate vascularization within solid tumors frequently results in hypoxia [14], a key driver of altered tumor biology. It is increasingly recognized that HRMs play a pivotal role in mediating cellular survival under hypoxic stress and in tumor progression. The present study offers the first comprehensive characterization of HRMs in canine HCC. Crucially, our observations indicate that HRM regulation in canine HCC reflects complementary regulatory patterns, encompassing both tumor-adaptive and stress-limiting responses, thereby providing new insight into the complexity of hypoxic tumor adaptation.

Hypoxic exposure resulted in significantly higher miRNA mapping rates than those seen with normoxia, indicating selective activation of miRNA expression under hypoxic conditions. Consistent with this observation, previous studies have identified miRNAs as one of the most prominently and consistently induced classes of non-coding RNAs during hypoxia [15,16,41]. Annotation of HRMs using both canine and human miRNA references revealed that a substantial proportion of differentially expressed miRNAs were identified exclusively through the human database, including 33.05% (upregulated) and 48.63% (downregulated) in 95-1044 cells and 34.55% and 55.26%, respectively, in 95-0112 cells. These findings highlight the current under-annotation of the canine miRNome, as miRBase lists far fewer mature miRNAs for dogs than for humans [42]. Given the evolutionary conservation of miRNAs across mammals, incorporation of human homologues enhances discovery in canine studies. However, such annotations require cautious interpretation, as not all human miRNAs represent true functional orthologs, underscoring the importance of experimental validation.

Through integrative analysis of cell line models, clinical tumor tissues, and circulating compartments, we identified a subset of HRMs consistently dysregulated across experimental systems. Among these, miR-210 and miR-34a emerged as the most robust and conserved candidates, supporting their biological relevance in hypoxia-associated tumor processes and highlighting their potential as circulating biomarkers.

miR-210 is widely regarded as a canonical hypoxia-responsive miRNA and a master regulator of the hypoxic response, robustly upregulated by hypoxia-inducible factors (HIFs) under low oxygen conditions [20]. Its conserved role in hypoxia adaptation is supported across multiple canine tumor types, including glioma [43], oral melanoma [44], and mammary neoplasia [45], as well as in exosomal compartments of oral melanoma and HCC [46]. Consistently, in human HCC, hypoxia-induced miR-210 promotes tumor progression by enhancing cell migration and invasion [47] and by driving metabolic reprogramming and angiogenesis [48], underscoring its conserved role in hypoxia-driven tumor progression across species. Accordingly, our observation of elevated cfa-miR-210 in hypoxia-exposed canine HCC cell lines is supportive of a hypoxia-responsive phenotype under the experimental conditions used, although classical hypoxia markers, such as HIF-1α, CAIX, GLUT1, were not directly evaluated.

While miR-34a is classically described as a p53-responsive, tumor-suppressive miRNA often downregulated in canine osteosarcoma and various human cancers [49,50,51,52,53,54,55], we observed its consistent upregulation across hypoxia-exposed canine HCC cells, clinical liver tissues, and plasma EVs. Similar context-dependent elevations of miR-34a have been documented in canine large B-cell lymphoma and distinct canine HCC cohorts [18,56].

This apparent discrepancy highlights the complex, context-dependent regulation of miR-34a, which may be influenced by a dynamic balance among TP53-dependent stress signaling, hypoxia-related pathways, and epigenetic regulation [57,58,59]. Because TP53 status, HIF activity, promoter methylation, and downstream stress-response or apoptotic markers were not evaluated in the present study, the precise molecular mechanism driving this upregulation remains to be determined.

Nevertheless, to assess the cross-species validity of this response, we interrogated two independent human HCC datasets (TCGA-LIHC and GSE21362). Notably, hsa-miR-34a-5p demonstrated reproducible upregulation across both human cohorts, supporting the translational relevance of miR-34a dysregulation in HCC. In contrast, while miR-210 showed robust hypoxia-induced elevation in canine HCC, it did not exhibit significant differential expression in either human dataset. This cross-species discrepancy may reflect differences in cohort composition, tumor heterogeneity, disease etiology, or the degree of hypoxia within the analyzed tissues. Further studies using well-characterized human HCC cohorts with defined hypoxic status are warranted to clarify the translational relevance of hypoxia-induced miR-210 expression.

Although hypoxia-mediated suppression of miR-34a has been linked to EMT and cell survival in certain contexts [57,60], its hypoxia-induced elevation can also trigger apoptosis and suppress autophagy in human HCC [61,62], alongside exerting potent antitumor activity in canine cancer models [49,53,63]. In the present study, the higher expression of miR-34a in clinical liver tissues than in hypoxia-exposed cell lines suggests that additional in vivo microenvironmental factors, such as inflammation and metabolic stress may contribute to its regulation [35,36,64]. Collectively, these findings suggest that miR-34a represents a context-dependent hypoxia-associated miRNA with potential translational relevance in HCC, although the molecular mechanisms underlying its differential regulation require further investigation.

With respect to clinical translation, these results highlight miR-210 and miR-34a as highly promising, minimally invasive biomarkers in canine HCC. miR-210 was robustly elevated in both plasma and plasma-derived EVs, consistent with its role as a systemic hypoxia marker, while miR-34a showed selective enrichment in plasma-derived EVs, suggesting enhanced tumor specificity. These observations support the potential application of a combined miR-210/miR-34a liquid biopsy panel for the detection and monitoring of canine HCC. More broadly, hypoxia-associated miRNA signatures may serve as indicators of tumor hypoxic status, with potential implications for prognosis and therapeutic stratification.

Integrated target prediction analyses suggested that miR-210 and miR-34a may putatively converge on major oncogenic pathways, including MAPK, Ras, and PI3K-Akt signaling, which are central to hepatocellular carcinoma progression [65]. Notably, TGIF2 and SPRED1 emerged as shared candidate targets, suggesting hypothetical coordinated regulation of tumor adaptation under hypoxic stress. The interactions between miR-210/TGIF2 and miR-34a/TGIF2 have been experimentally validated in previous studies, demonstrating their involvement in cell-cycle control and metastatic suppression [66,67]. Likewise, the regulation of SPRED1 by miR-210 has been previously reported [68]. However, these putative interactions remain computationally predicted in canine HCC. Therefore, direct experimental validation (e.g., dual-luciferase assays and miRNA gain- or loss-of-function studies) is essential to confirm whether these regulatory networks operate in canine HCC and to define their precise molecular mechanisms.

From a comparative oncology perspective, canine HCC represents a highly relevant model for human disease, as tumors arise spontaneously in an intact immune system and under natural environmental exposures, better reflecting tumor heterogeneity and tumor-host interactions than induced rodent models [7]. Strong genomic and molecular conservation between dogs and humans further supports translational relevance [69,70]. Thus, identifying conserved, microenvironment-responsive molecular regulators in canine HCC may offer valuable insights into the mechanisms underlying human HCC progression.

Among such regulators, miR-210 and miR-34a are evolutionarily conserved, hypoxia-responsive miRNAs whose roles in canine HCC remain largely uncharacterized. While we did not experimentally establish miR-34a’s function in the canine context, cross-species analysis of its human homolog (hsa-miR-34a-5p) revealed canonical tumor-suppressive features, including an inverse correlation with oncogenic targets and an association with improved patient survival. This suggests miR-34a likely exerts a similar stress-limiting role in dogs. Conversely, the well-established hypoxia-responsive miR-210, regulated by HIF-1α, correlates with poor clinical outcomes in human HCC, marking it as a driver of hypoxia-mediated aggressiveness. Collectively, these findings suggest that, despite evolutionary conservation, hypoxia-responsive miRNAs may exhibit functionally divergent yet complementary roles, with miR-210 primarily reflecting adaptive tumor progression and miR-34a potentially contributing to stress-limiting regulatory mechanisms. While the tumor-suppressive function of miR-34a in canine HCC remains to be experimentally validated, the concordant human data provide a strong rationale for its investigation as a candidate regulator of tumor restraint in comparative oncology models.

Despite providing the first comprehensive characterization of hypoxia-responsive miRNAs in canine HCC, this study has several limitations. Classical hypoxia markers were not experimentally evaluated, and gain- or loss-of-function assays and direct validation of predicted miRNA–mRNA interactions were beyond the scope of this study. In addition, the molecular mechanisms underlying miR-34a upregulation, including the potential involvement of TP53 status, HIF signaling, and epigenetic regulation, were not investigated. Although the plasma-derived EVs analyzed in this study had previously been evaluated for the EV-associated protein markers CD9 and TSG101, full MISEV-compliant characterization was not performed. Furthermore, the relatively small clinical cohort and the reliance on public human datasets rather than parallel experiments in human HCC cell lines warrant further validation. Future studies incorporating side-by-side hypoxic exposure experiments in both human and canine HCC cell lines, together with larger independent clinical cohorts, will strengthen the comparative and translational significance of these findings.

## 5. Conclusions

This study identifies miR-210 and miR-34a as potential complementary hypoxia-responsive regulators in canine HCC, suggesting conserved adaptive and stress-limiting programs within the hypoxic tumor microenvironment. Their consistent dysregulation across experimental and clinical datasets, together with their enrichment in EVs, highlights their potential as minimally invasive biomarkers for hypoxia-associated HCC. These findings further suggest that HRMs programs may contribute to molecular stratification of hepatocellular carcinoma based on hypoxic status, while targeting hypoxia-associated miRNA networks may represent a promising future therapeutic strategy in HCC.

## Data Availability

The canine datasets generated and analyzed during the current study have been deposited in the NCBI BioProject database under accession number PRJNA1464337 (https://www.ncbi.nlm.nih.gov/sra/PRJNA1464337; accessed 10 May 2026). Additional data supporting the findings of this study are available within the article and its Supplementary Materials.

## Supplementary Materials

The following supporting information can be downloaded at: https://www.mdpi.com/article/10.3390/cells15151341/s1, Figure S1: Read length distribution of NGS libraries from canine HCC cell lines under normoxic and hypoxic conditions; Figure S2: Hierarchical clustering of differentially expressed miRNAs in canine HCC cell lines under hypoxic and normoxic conditions; Figure S3: Venn diagram analysis of hypoxia-responsive miRNAs annotated to canine and human miRBase references in canine HCC cell lines; Figure S4: Expression profiles of eight hypoxia-responsive miRNAs consistently upregulated in canine HCC clinical tissues and cell lines under hypoxic and normoxic conditions. The expression levels of eight hypoxia-responsive miRNAs, identified as consistently upregulated in our small RNA-seq datasets, are presented across two canine HCC cell lines (95-1044 and 95-0112) and clinical HCC tissues; Figure S5: Expression profiles of three hypoxia-responsive miRNAs consistently downregulated in canine HCC clinical tissues and cell lines under hypoxic and normoxic conditions. The expression levels of three hypoxia-responsive miRNAs, identified as consistently downregulated in our small RNA-seq datasets, are presented across two canine HCC cell lines (95-1044 and 95-0112) and clinical HCC tissues; Figure S6: qPCR validation of hypoxia-responsive miRNAs in canine HCC cells (95-1044 and 95-0112), clinical tissues, and circulating compartments; Figure S7: Identification of high-confidence predicted target genes of cfa-miR-210 and cfa-miR-34a using multiple miRNA target prediction databases in canine HCC; Figure S8: Sequence conservation of candidate hypoxia-responsive miRNAs between canine and human; Figure S9: Expression correlation analysis of canine-derived hypoxia-responsive miRNAs and their predicted shared candidate target genes in human HCC (TCGA-LIHC); Figure S10: Expression analysis of human homologs of candidate canine hypoxia-responsive miRNAs in human HCC datasets; Figure S11: Prognostic significance of human homologs of candidate canine hypoxia-responsive miRNAs based on Kaplan-Meier overall survival (OS) analyses in human HCC using TCGA-LIHC datasets; Table S1. Details of samples included in this study; Table S2. Sample-wise summary of total, mapped, and unmapped reads and their percentages in RNA-Seq data from two canine HCC cell lines (95-1044 and 95-0112); Table S3. Differentially expressed microRNAs (DE miRNAs) identified in hypoxic 95-1044 canine HCC cells (*n* = 3) compared with normoxic controls (*n* = 3) using NGS analysis; Table S4. Differentially expressed microRNAs (DE miRNAs) identified in hypoxic 95-0112 canine HCC cells (*n* = 3) compared with normoxic controls (*n* = 3) using NGS analysis; Table S5. A list of differentially expressed miRNAs in canine HCC clinical tissues (*n* = 3) compared with normal liver tissues (*n* = 3); Table S6. Bioinformatic prediction of candidate target genes for cfa-miR-210 and cfa-miR-34a in canine HCC; Table S7. List of high-confidence predicted target genes (*n* = 154) for the hypoxia-responsive miRNAs, cfa-miR-210 and cfa-miR-34a; Table S8. Gene Ontology (GO) terms enriched from 154 high-confidence predicted target genes of cfa-miR-210 and cfa-miR-34a in canine HCC, identified using DAVID; Table S9. KEGG pathways enriched from 154 high-confidence predicted target genes of cfa-miR-210 and cfa-miR-34a in canine HCC, identified using DAVID. Table S10. Normalized expression values of hsa-miR-34a and hsa-miR-210 in paired human HCC and normal liver tissues from the GSE21362 dataset.

## Abbreviations

The following abbreviations are used in this manuscript:

AUC	Area Under the Curve
DE	Differentially Expressed
EMT	Epithelial-to-Mesenchymal Transition
ENCORI	Encyclopedia of RNA Interactomes
EVs	Extracellular Vesicles
FDR	False Discovery Rate
GO	Gene Ontology
GEO	Gene Expression Omnibus
HCC	Hepatocellular Carcinoma
HIF	Hypoxia-Inducible Factor
HRM	Hypoxia-Responsive microRNA
KEGG	Kyoto Encyclopedia of Genes and Genomes
LIHC	Liver Hepatocellular Carcinoma
MAPK	Mitogen-Activated Protein Kinase
NCBI	National Center for Biotechnology Information
NGS	Next-Generation Sequencing
OS	Overall Survival
PCA	Principal Component Analysis
PI3K	Phosphoinositide 3-Kinase
qPCR	Quantitative Polymerase Chain Reaction
Rap1	Ras-Related Protein 1
Ras	Rat Sarcoma Viral Oncogene Homolog
ROC	Receiver Operating Characteristic
RT-qPCR	Reverse Transcription Quantitative Polymerase Chain Reaction
SPRED1	Sprouty-Related EVH1 Domain-Containing Protein 1
TCGA	The Cancer Genome Atlas
TGIF2	TGFB-Induced Factor Homeobox 2
TME	Tumor Microenvironment
