# Supplementary material for: Conserved Hypoxia-Responsive miRNA Programs Define Adaptive and Stress-Limiting Regulatory Axes in Hepatocellular Carcinoma
Source: Cells. 2026 Jul 26;15(15):1341. doi: 10.3390/cells15151341 (PMC13465076; doi:10.3390/cells15151341)
Supplement: Supplementary file 1 [file cells-15-01341-s001.zip › Spplementary figures_V3_without track change.pdf]

Conserved hypoxia-responsive miRNA programs define adaptive and stress-limiting regulatory axes in hepatocellular carcinoma

Most Shumi Akhter Shathi[1], Mohammad Arif[1,2], Nobuhiro Nozaki[1], MD Nazmul Hasan[1], Yutaro Ide[1], Yoshiyuki Akiyama[1], Shaohsu Wang[1], Sirazul Islam[1,3], Tanjila Rahman[1,4], Tomohide Kuramoto[5], Yu Furusawa[5], Takeshi Sogawa[5], Kaori Takahashi[5], Aki Noguchi[5], Tatsuro Hifumi[1,6], Shinji Hirano[1,6], Noriaki Miyoshi[1,6], Osamu Yamato[1,5], Masashi Takahashi[1,5] and Naoki Miura[1,5*]

1 Joint Graduate School of Veterinary Medicine, Kagoshima University, Kagoshima 890-8580, Japan
2 Department of Microbiology and Hygiene, Bangladesh Agricultural University, Mymensingh 2202, Bangladesh
3 Department of Livestock Services (DLS), Farmgate, Dhaka 1215, Bangladesh
4 Department of Anatomy, Histology & Physiology, Sher-e-Bangla Agricultural University, Dhaka 1207, Bangladesh
5 Veterinary Teaching Hospital, Joint Faculty of Veterinary Medicine, Kagoshima University, Kagoshima 890-8580, Japan
6 Department of Veterinary Pathology, Joint Faculty of Veterinary Medicine, Kagoshima University, Kagoshima 890-8580, Japan
* **Correspondence**: k9236024@kadai.jp; Tel.: +81-99-285-3527

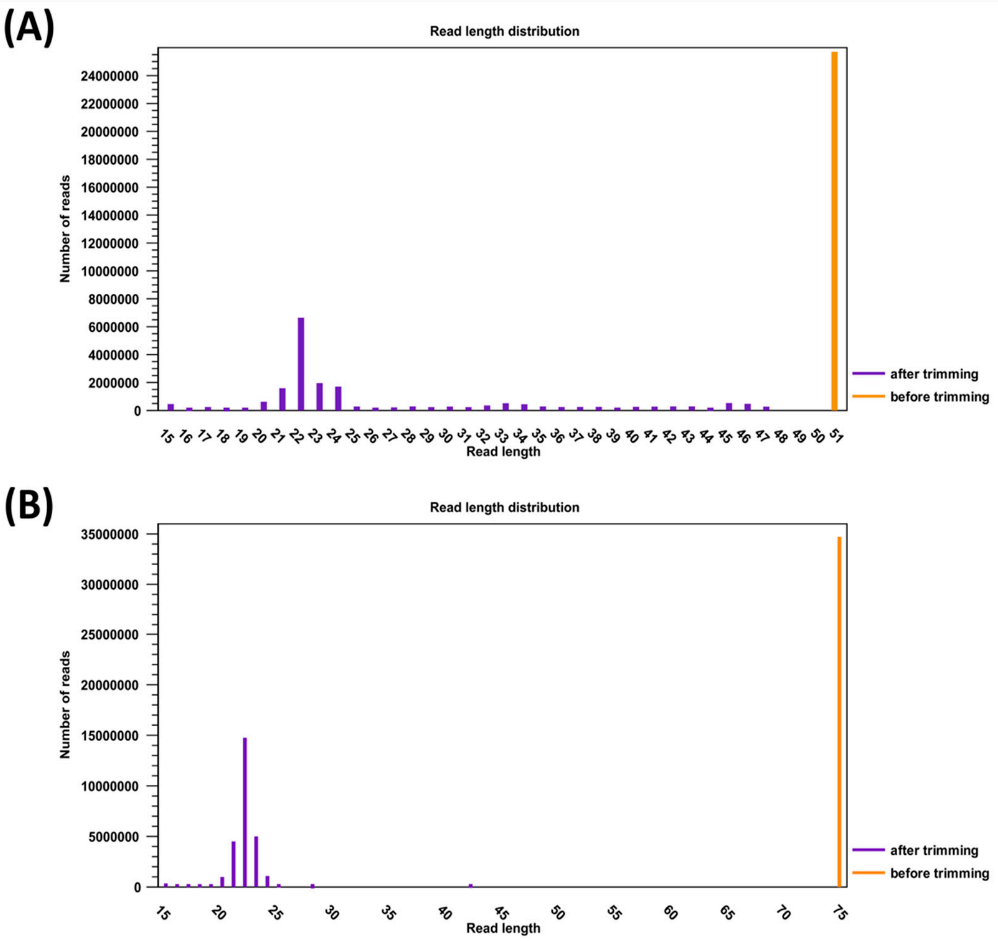

Figure S1. Read length distribution of NGS libraries from canine HCC cell lines under normoxic and hypoxic conditions. **(A)** Representative read length distribution for the normoxia group. **(B)** Representative read length distribution for the hypoxia group. In both panels, the orange bars indicate read lengths before trimming, while the purple bars indicate read lengths after trimming. The x-axis represents read length (bp), and the y-axis represents the number of reads. Trimming effectively removes adapter and low-quality bases, resulting in a shift toward shorter, more uniform read lengths compared with the untrimmed libraries.

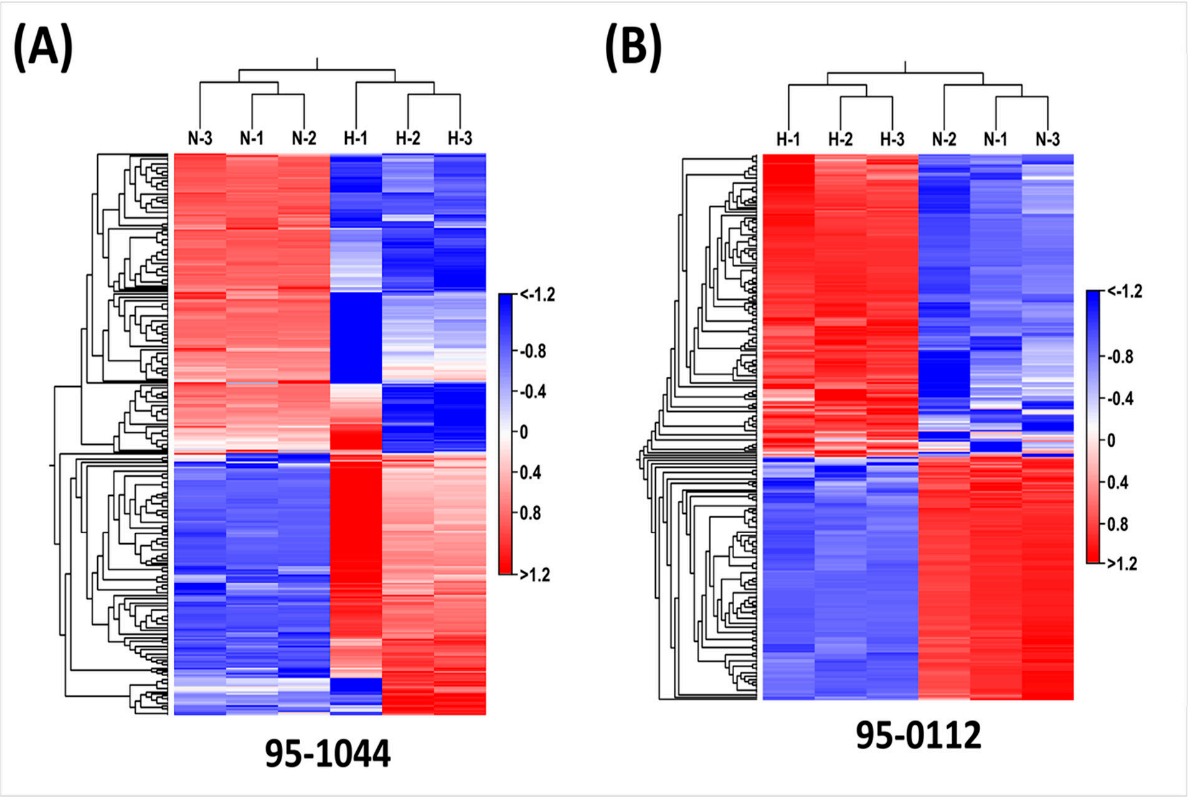

Figure S2. Hierarchical clustering of differentially expressed miRNAs in canine HCC cell lines under hypoxic and normoxic conditions. **(A)** Heatmap showing hierarchical clustering of hypoxia-responsive miRNAs in the 95-1044 canine HCC cell line. **(B)** Heatmap showing hierarchical clustering of hypoxia-responsive miRNAs in the 95-0112 canine HCC cell line. For each panel, miRNA expression profiles from hypoxic (H-1, H-2, H-3) and normoxic (N-1, N-2, N-3) samples are shown, with rows representing differentially expressed miRNAs and columns representing biological replicates. In the 95-1044 cell line, a total of 332 hypoxia-responsive miRNAs were identified, including 154 upregulated and 178 downregulated miRNAs, whereas in the 95-0112 cell line, 321 hypoxia-responsive miRNAs were identified, including 178 upregulated and 143 downregulated miRNAs, compared with their respective normoxic controls. Color intensity reflects relative expression levels (row-scaled), with red indicating higher expression and blue indicating lower expression.

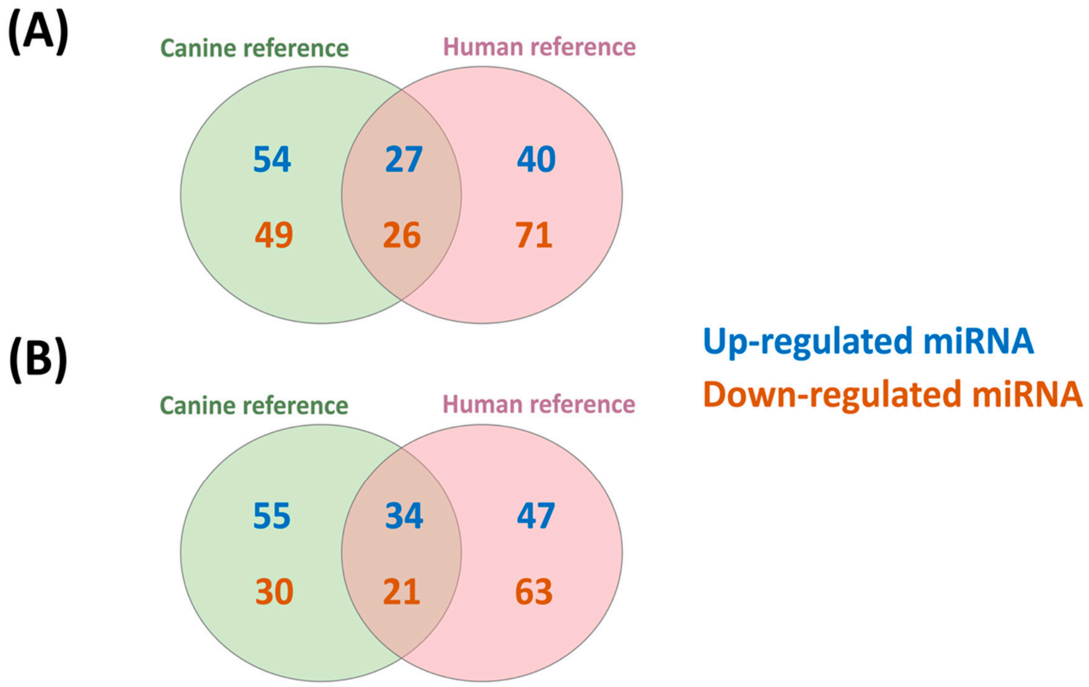

Figure S3. Venn diagram analysis of hypoxia-responsive miRNAs annotated to canine and human miRBase references in canine HCC cell lines. **(A)** Venn diagram showing the overlap of differentially expressed miRNAs identified in hypoxia versus normoxia in the 95-1044 canine HCC cell line. **(B)** Venn diagram showing the corresponding analysis in the 95-0112 cell line. In each plot, the green circle represents miRNAs annotated using the canine (dog) reference, while the red circle represents miRNAs annotated using the human miRBase reference. Numbers shown in blue indicate up-regulated miRNAs, whereas numbers shown in orange indicate down-regulated miRNAs under hypoxic conditions compared with normoxia. Overlapping regions denote miRNAs commonly annotated in both canine and human references, while non-overlapping regions represent species-specific annotations. These analyses highlight both conserved and species-specific hypoxia-responsive miRNA signatures in canine HCC cells.

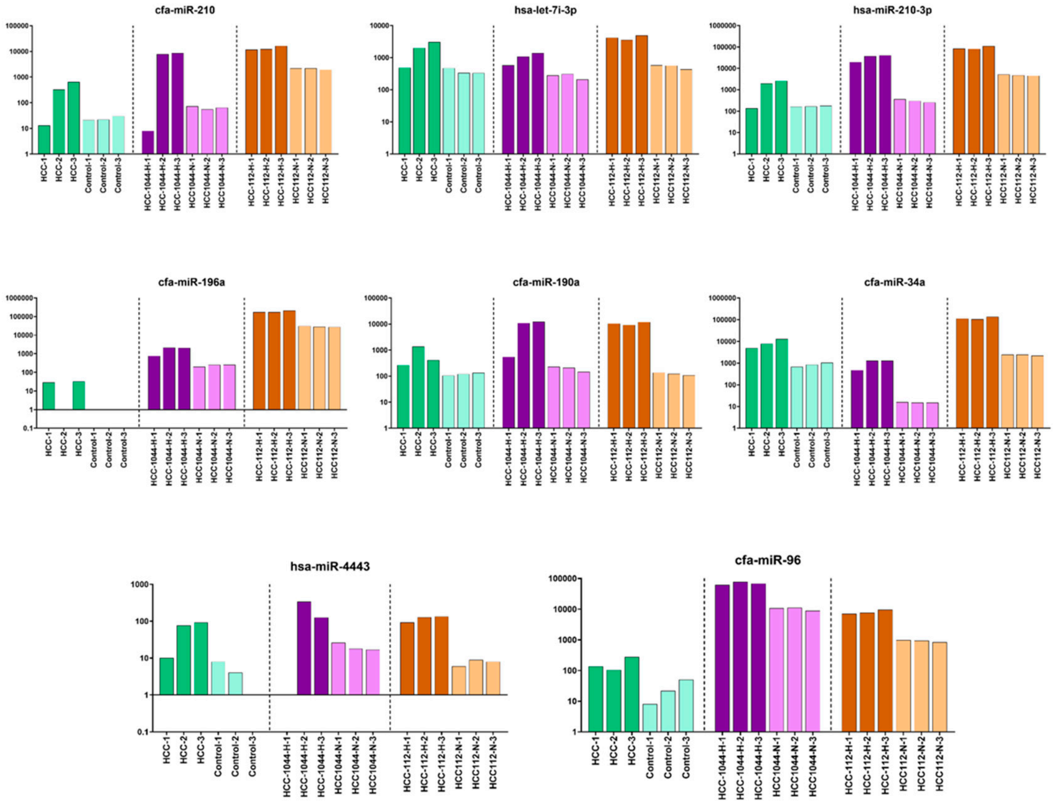

Figure S4. Expression profiles of eight hypoxia-responsive miRNAs consistently upregulated in canine HCC clinical tissues and cell lines under hypoxic and normoxic conditions. The expression levels of eight hypoxia-responsive miRNAs, identified as consistently upregulated in our small RNA-seq datasets, are presented across two canine HCC cell lines (95-1044 and 95-0112) and clinical HCC tissues. Bar plots represent normalized miRNA expression values derived from next-generation sequencing data and analyzed using CLC Genomics Workbench v24. Liver samples include HCC tissues (HCC-1 to HCC-3) and non-tumor controls (Control-1 to Control-3). Cell line data are shown for 95-1044 (HCC-1044) and 95-0112 (HCC-112) under hypoxic (H) and normoxic (N) conditions, with three biological replicates per condition. The y-axis indicates expression values for each miRNA in a log10 scale, based on the maximum group mean across clinical tissues and cell line samples to facilitate comparative visualization. Dashed vertical lines separate clinical tissue samples from cell line datasets and lighter and deeper color distinguished groups in each sample type. Each panel corresponds to an individual miRNA.

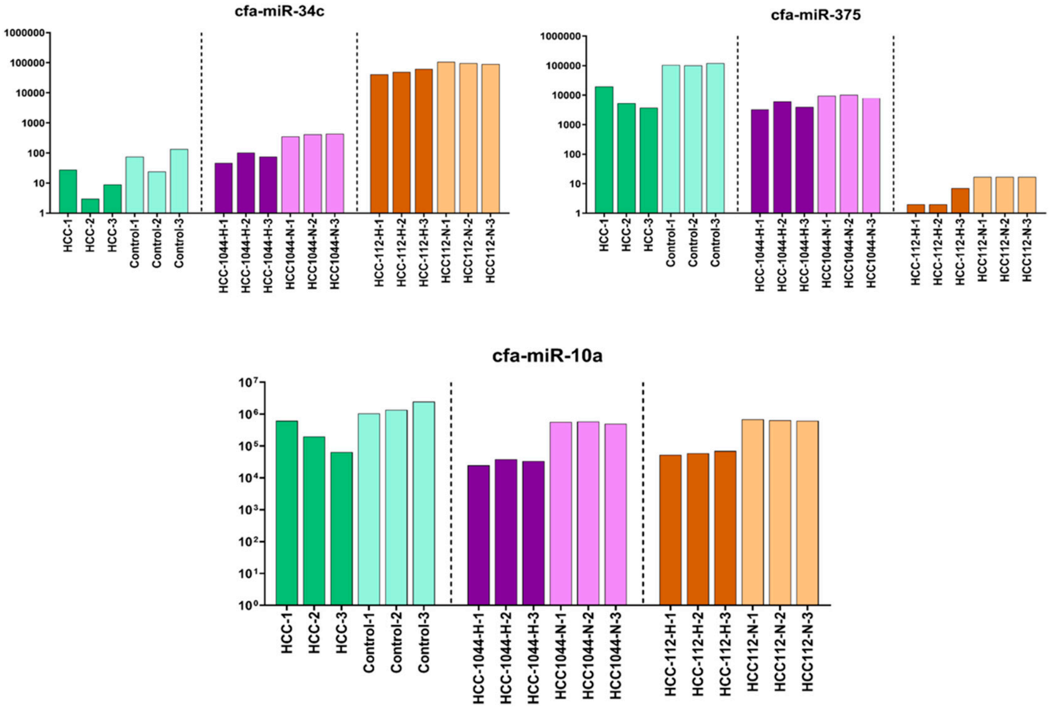

Figure S5. Expression profiles of three hypoxia-responsive miRNAs consistently downregulated in canine HCC clinical tissues and cell lines under hypoxic and normoxic conditions. The expression levels of three hypoxia-responsive miRNAs, identified as consistently downregulated in our small RNA-seq datasets, are presented across two canine HCC cell lines (95-1044 and 95-0112) and clinical HCC tissues. Bar plots represent normalized miRNA expression values derived from next-generation sequencing data and analyzed using CLC Genomics Workbench v24. Clinical samples include HCC tissues (HCC-1 to HCC-3) and non-tumor controls (Control-1 to Control-3). Cell line data are shown for 95-1044 (HCC-1044) and 95-0112 (HCC-112) under hypoxic (H) and normoxic (N) conditions, with three biological replicates per condition. The y-axis indicates expression values for each miRNA in a log10 scale, based on the maximum group mean across clinical tissues and cell line samples to facilitate comparative visualization. Dashed vertical lines separate clinical tissue samples from cell line datasets and lighter and deeper color distinguished groups in each sample type. Each panel corresponds to an individual miRNA.

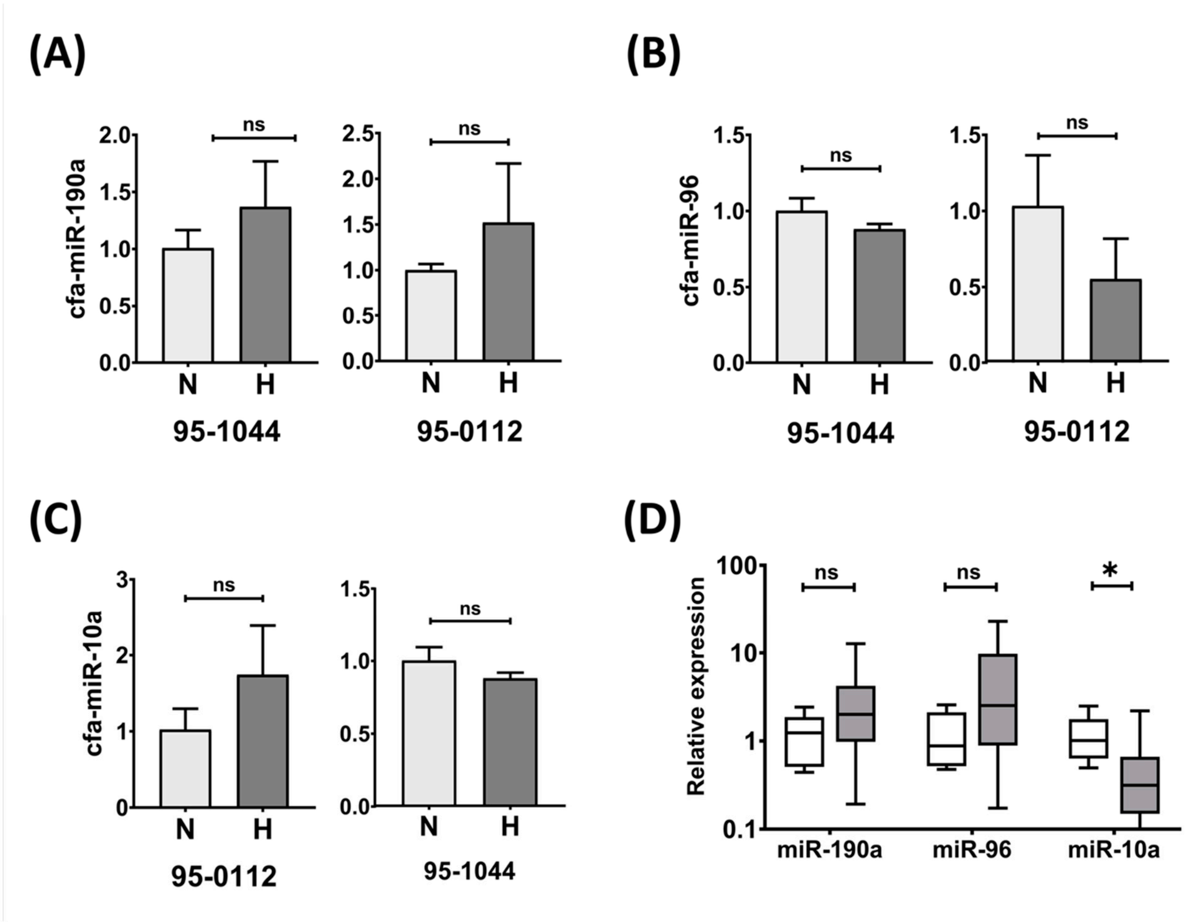

Figure S6. qPCR validation of hypoxia-responsive miRNAs in canine HCC cells (95-1044 and 95-0112), clinical tissues, and circulating compartments. **(A)** Relative expression of cfa-miR-190a measured by RT-qPCR in canine HCC cell lines 95-1044 (left) and 95-0112 (right) following hypoxic exposure, normalized to RNU6B (n = 3 per group). Here, N denotes to normoxic and H denotes hypoxic samples. **(B)** Relative expression of cfa-miR-196 in 95-1044 (left) and 95-0112 (right) cells under hypoxic conditions, normalized to RNU6B (n = 3 per group). **(C)** Relative expression of cfa-miR-10a in 95-1044 (left) and 95-0112 (right) cells under hypoxic conditions, normalized to RNU6B (n = 3 per group). **(D)** Relative expression levels of cfa-miR-190a, cfa-miR-96 and cfa-miR-10a in canine HCC clinical tissues (n = 12) compared with normal liver controls (n = 5), plotted on a log10 scale. Data are presented as mean ± SD. Statistical significance was assessed using an unpaired two-tailed Student's t-test for normally distributed data or the Mann-Whitney U test for non-normally distributed data. A p-value < 0.05 was considered statistically significant (∗, $p < 0.05$; ns, not significant).

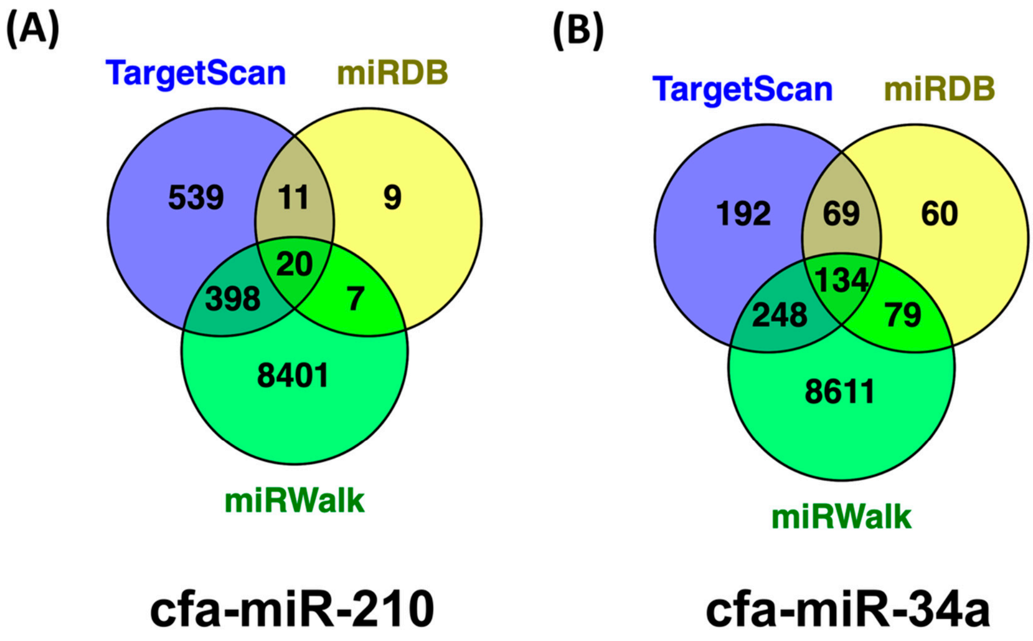

Figure S7. Identification of high-confidence predicted target genes of cfa-miR-210 and cfa-miR-34a using multiple miRNA target prediction databases in canine HCC. **(A-B)** Venn diagram analysis showing the overlap of predicted canine specific target genes for cfa-miR-210 (A) and cfa-miR-34a (B) identified using three independent miRNA target prediction databases: TargetScan, miRDB, and miRWalk. Predicted target genes were filtered to obtain high-confidence targets based on the following criteria: TargetScan (context++ score ≤ -0.15), miRDB (target score ≥ 60), and miRWalk (binding probability ≥ 0.9 and minimum free energy ≤ -20). Numbers within each Venn diagram indicate the total number of predicted targets unique to each database or shared among databases. Genes common to all three databases were considered high-confidence predicted targets and used for downstream analyses.

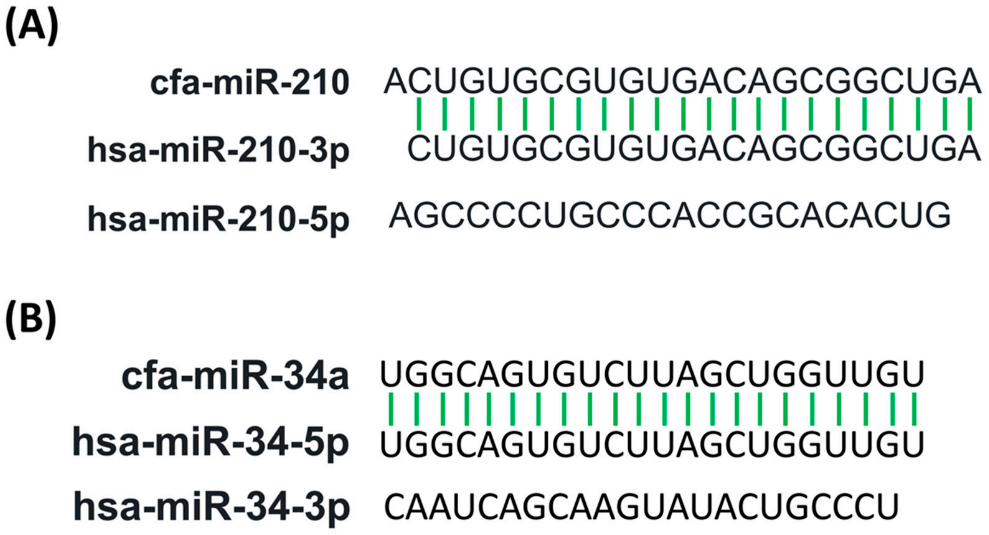

Figure S8. Sequence conservation of candidate hypoxia-responsive miRNAs between canine and human. Multiple sequence alignment showing nucleotide sequence conservation between canine and human miRNAs. Conservation is illustrated for **(A)** miR-210 (cfa-miR-210 vs. hsa-miR-210-3p) and **(B)** miR-34a (cfa-miR-34a vs. hsa-miR-34-5p).

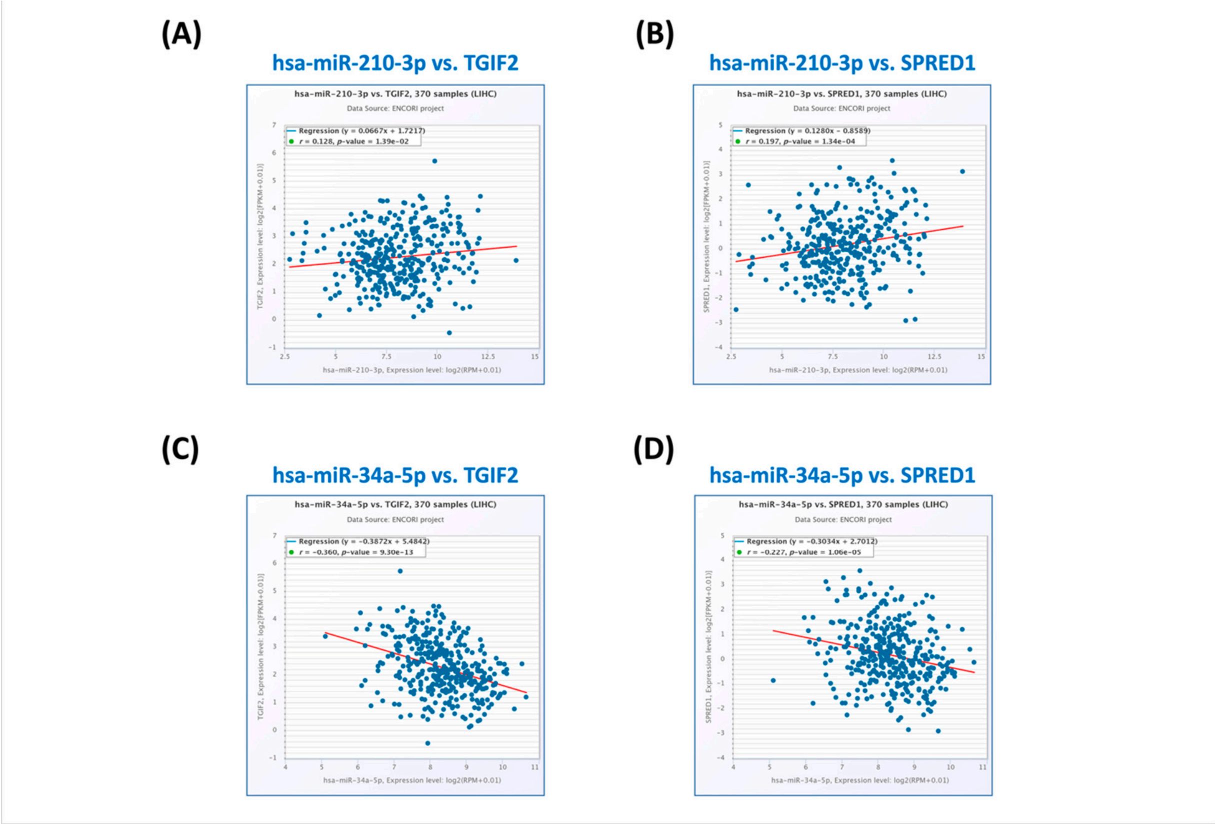

Figure S9. Expression correlation analysis of canine-derived hypoxia-responsive miRNAs and their predicted shared candidate target genes in human HCC (TCGA-LIHC). **(A-B)** Correlation analysis between the human homolog of the canine hypoxia-responsive miRNA cfa-miR-210 (hsa-miR-210-3p) and its predicted target genes *TGIF2* (A) and *SPRED1* (B) in human HCC. **(C-D)** Correlation analysis between the human homolog of the canine hypoxia-responsive miRNA cfa-miR-34a (hsa-miR-34a-5p) and its predicted target genes *TGIF2* (C) and *SPRED1* (D) in human HCC. Scatter plots show expression correlations based on RNA-seq data from 370 TCGA-LIHC samples, obtained from the ENCORI database. Red lines indicate linear regression fits; Pearson correlation coefficients (r) and corresponding *p*-values are shown in each panel.

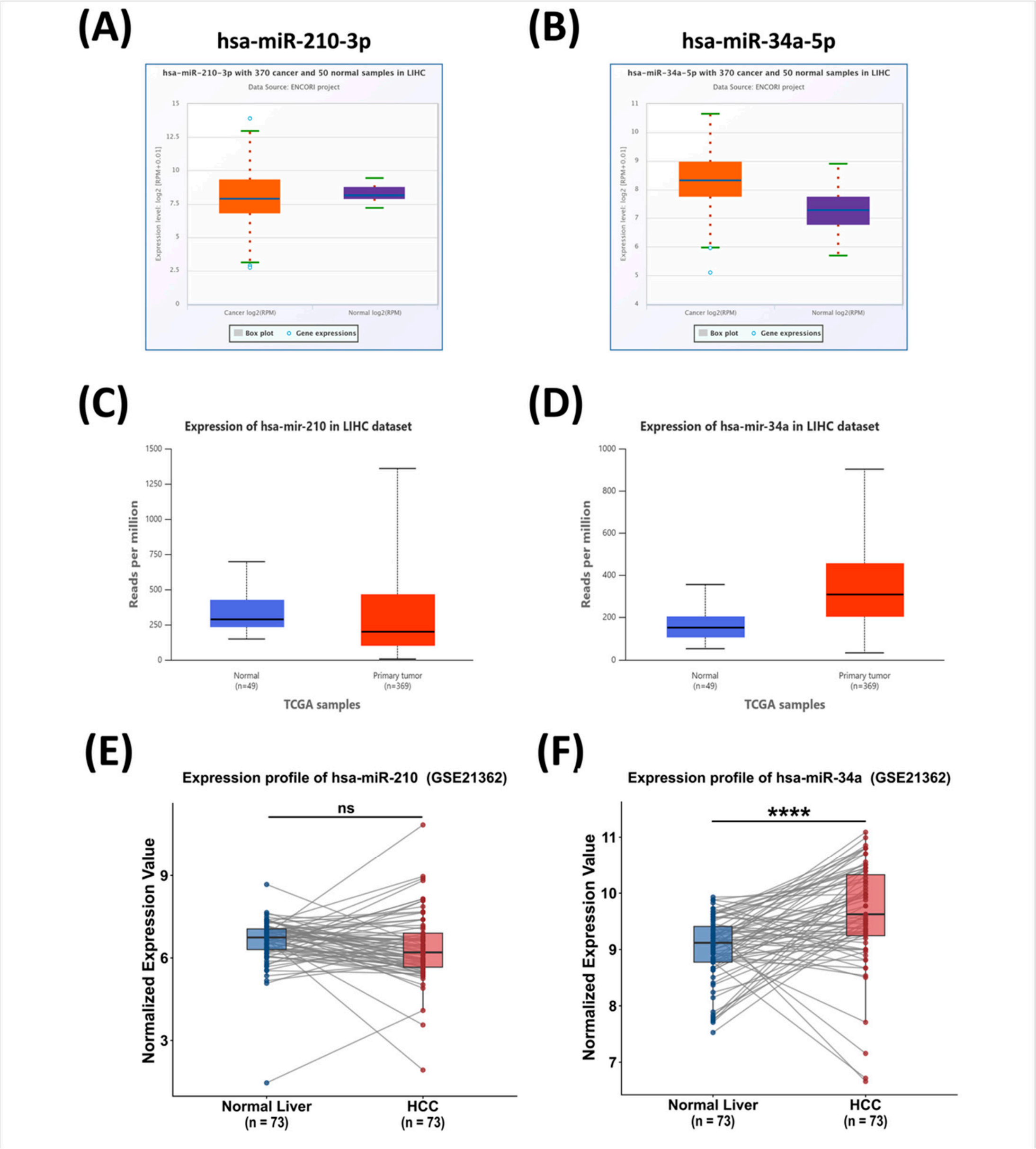

Figure S10. Expression analysis of human homologs of candidate canine hypoxia-responsive miRNAs in human HCC datasets. **(A-B)** Expression levels of hsa-miR-210-3p (human homolog of cfa-miR-210) (A) and hsa-miR-34a-5p (human homolog of cfa-miR-34a) (B) in human hepatocellular carcinoma (HCC) and normal liver tissues, analyzed using the ENCORI database. **(C-D)** Corresponding expression profiles of hsa-miR-210 (C) and hsa-miR-34a (D) analyzed using the UALCAN database. **(E–F)** Validation of miRNA expression in an independent microarray dataset (GSE21362) comprising paired human HCC and tumor-adjacent normal liver tissues (n = 73 matched pairs). Expression profiles of hsa-miR-210 (E) and hsa-miR-34a (F) are presented as paired line plots overlaid with box plots. Individual gray lines connect paired samples from the same patient. Statistical significance was evaluated using a paired two-tailed Student's t-test (**** $p < 0.0001$; ns = not significant, $p \geq 0.05$).

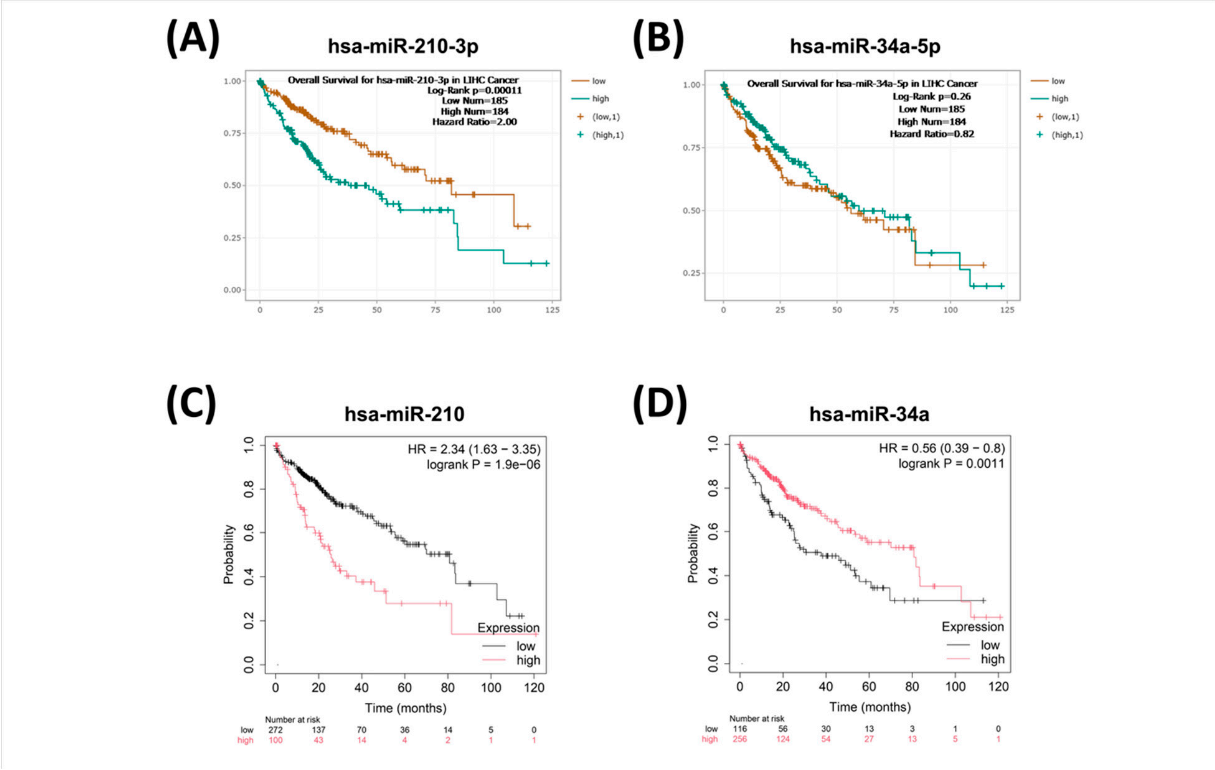

Figure S11. Prognostic significance of human homologs of candidate canine hypoxia-responsive miRNAs based on Kaplan-Meier overall survival (OS) analyses in human HCC using TCGA-LIHC datasets. **(A-B)** OS analysis of hsa-miR-210-3p (human homolog of cfa-miR-210) (A) and hsa-miR-34a-5p (human homolog of cfa-miR-34a) (B) using the ENCORI database. **(C-D)** OS analysis of hsa-miR-210 (C) and hsa-miR-34a (D) using the KM plotter database. Patients were stratified into high and low expression groups based on median expression levels. Log-rank *p*-values and hazard ratios (HR) are shown on each plot.
